# Characterization of ligand-receptor pair in acute myeloid leukemia: a scoring model for prognosis, therapeutic response, and T cell dysfunction

**DOI:** 10.3389/fonc.2024.1473048

**Published:** 2024-10-17

**Authors:** Chunlan Fu, Di Qiu, Mei Zhou, Shaobo Ni, Xin Jin

**Affiliations:** ^1^ Department of Hematology, Zhuji Affiliated Hospital of Wenzhou Medical University, Zhuji, Zhejiang, China; ^2^ Department of Breast Surgery, Zhuji Affiliated Hospital of Wenzhou Medical University, Zhuji, Zhejiang, China

**Keywords:** Ligand-receptor, Acute Myeloid Leukemia, prognosis, therapeutic response, T cell

## Abstract

**Introduction:**

The significance of ligand-receptor (LR) pair interactions in the progression of acute myeloid leukemia (AML) has been the focus of numerous studies. However, the relationship between LR pairs and the prognosis of AML, as well as their impact on treatment outcomes, is not fully elucidated.

**Methods:**

Leveraging data from the TCGA-LAML cohort, we mapped out the LR pair interactions and distinguished specific molecular subtypes, with each displaying distinct biological characteristics. These subtypes exhibited varying mutation landscapes, pathway characteristics, and immune infiltration levels. Further insight into the immune microenvironment among these subtypes revealed disparities in immune cell abundance.

**Results:**

Notably, one subtype showed a higher prevalence of CD8 T cells and plasma cells, suggesting increased adaptive immune activities. Leveraging a multivariate Lasso regression, we formulated an LR pair-based scoring model, termed “LR.score,” to classify patients based on prognostic risk. Our findings underscored the association between elevated LR scores and T-cell dysfunction in AML. This connection highlights the LR score’s potential as both a prognostic marker and a guide for personalized therapeutic interventions. Moreover, our LR.score revealed substantial survival prediction capacities in an independent AML cohort. We highlighted CLEC11A, ICAM4, ITGA4, and AVP as notably AML-specific.

**Discussion:**

qRT-PCR analysis on AML versus normal bone marrow samples confirmed the significant downregulation of CLEC11A, ITGA4, ICAM4, and AVP in AML, suggesting their inverse biomarker potential in AML. In summary, this study illuminates the significance of the LR pair network in predicting AML prognosis, offering avenues for more precise treatment strategies tailored to individual patient profiles.

## Introduction

Acute Myeloid Leukemia (AML) is a hematological malignancy characterized by the rapid growth of abnormal myeloid progenitor cells in the bone marrow and peripheral blood, accounting for approximately 20% of leukemia cases in adults (1), with a high degree of clinical and molecular heterogeneity. The prognosis for AML varies widely depending on factors such as age, cytogenetic abnormalities, and molecular mutations, with older patients and those experiencing relapse generally having poorer outcomes (2–5).

Recent advances in high-throughput sequencing technologies have significantly enhanced our understanding of the genetic and epigenetic landscapes of AML (6–8). These include mutations in genes such as NPM1, FLT3, DNMT3A, IDH1, and IDH2, which are frequently observed in AML and are associated with distinct clinical outcomes. Molecular classifiers have been developed to categorize AML patients into risk-specific subgroups, including cytogenetically normal AML (CN-AML), core-binding factor AML (CBF-AML), and subgroups defined by specific mutations like FLT3-ITD or IDH mutations (9–13). These classifications have proven valuable in predicting prognosis and guiding therapeutic decisions. Despite these advances, the prognosis for AML remains relatively poor, particularly in older patients and those who relapse post-treatment.

Despite these advances, AML remains a challenging disease to treat, particularly due to its complex tumor microenvironment (TME). The TME in AML is composed of a variety of cell types, including leukemic cells, stromal cells, immune cells, and the extracellular matrix, all of which interact to influence disease progression and response to therapy (14–16). The interactions between leukemic cells and their surrounding stroma are mediated by a network of ligand-receptor (LR) pairs that facilitate cell-cell communication, migration, and survival. While individual LR pairs, such as CXCL12/CXCR4, have been studied extensively in AML, the broader network of LR interactions and its impact on disease biology remains poorly understood.

In this study, we employed advanced computational approaches to map the LR pair interaction network in AML and to correlate these interactions with clinical outcomes. By analyzing transcriptomic data from large AML cohorts, we identified three distinct molecular subtypes characterized by unique LR pair-related gene signatures. These subtypes exhibit different mutation landscapes, pathway activation profiles, and levels of immune cell infiltration. Our LR pair-based scoring system, LR.score, demonstrated significant potential in predicting patient responses to both traditional chemotherapy and emerging targeted therapies, with a particular emphasis on its association with T-cell dysfunction and the downregulation of specific markers such as CLEC11A, ICAM4, ITGA4, and AVP in AML. These findings provide new insights into the complexity of cell-cell communication in AML and offer a robust framework for developing personalized treatment strategies tailored to the molecular characteristics of individual patients.

## Materials and methods

### Data acquisition and processing

**Figure 1:**
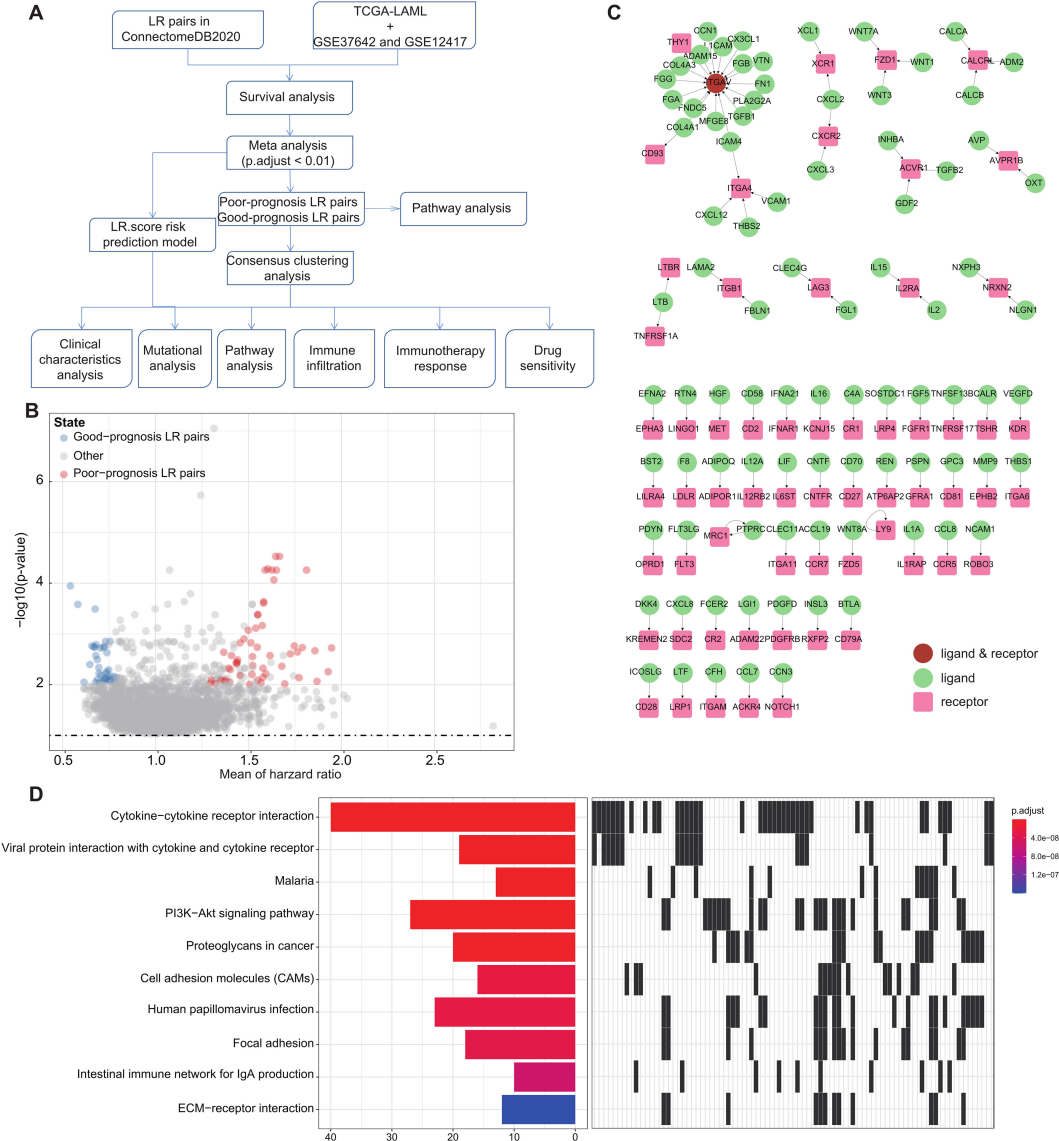
LR pairs with prognostic significance **(A)**. Overview of the study’s workflow **(B)**. Volcano plot representations highlight LR pairs with significant prognostic implications. LR genes associated with favorable prognosis are denoted in red, while those linked to poor prognosis are illustrated in blue **(C)**. Network visualization of the significant prognostic LR pairs, with receptors showcased in red and ligands in green **(D)**. The top ten enriched KEGG pathways derived from the prognostic-relevant LR pairs are presented.

We sourced clinical and RNA-Seq data specific to Acute Myeloid Leukemia (AML) from the TCGA dataset, accessible through the UCSC Xena portal (https://xenabrowser.net). Each tumor’s gene expression data was aligned with the human hg38 genome annotation. For subsequent analyses, we converted the gene expression measurements to Transcripts Per Kilobase Million (TPM) followed by log2 transformation ([TPM] + 1). We excluded samples that were missing either gene expression details or clinical annotations, resulting in a primary cohort of 142 AML patients. For our validation efforts, we turned to two public datasets: GSE37642 (17–20) and GSE12417 (21, 22). We incorporated only the primary AML samples with available clinical details and normalized gene expression metrics. We omitted probes without gene annotation, and for genes associated with multiple probes, the median expression was determined. This process led to the creation of validation sets with 417 and 163 AML samples, respectively. Additionally, we integrated 2293 Ligand-Receptor (LR) pairs from the connectome DB2020 database (23) detailed in (**Supplementary Table S1**). A schematic outline of our research methodology is presented in (**Figure 1A**).

### Stratification and survival analysis of patients based on ligand-receptor pairs

For each set of patient data, individuals were grouped into “high” or “low” categories based on the aggregate expression levels of specific ligand-receptor (LR) gene pairs. A patient was labeled as “high” if their total LR pair gene expression met or exceeded the median value for the entire patient group; otherwise, they were designated as “low.” To assess the influence of these classifications on patient outcomes, we utilized R’s “Survival” package (version 4.3.2). The log-rank test was employed to establish statistical significance, and hazard ratios (HRs) were derived using Cox regression models. Each patient group’s survival outcomes were scrutinized independently. To amalgamate insights from various cohorts, we turned to a meta-analysis approach, deploying the “Edgington” method through the “sump” function in R’s “metap” package (version 1.4). We selected the 94 significant LR pairs based on two primary criteria: 1) a Storey-adjusted q-value less than 0.1, ensuring statistical significance across multiple hypothesis tests, and 2) a consistent hazard ratio, either surpassing or falling below 1, across all evaluated cohorts, indicating a robust association with patient survival outcomes. To adjust for multiple hypothesis testing, we adopted Storey’s method (24) via the “qvalue” package in R (version 2.18.0).

### Samples clustering through consensus clustering

Using consensus clustering, we categorized the samples based on their gene expression patterns. We generated a consistency matrix with the “ConsensusClusterPlus” package in R (25). After narrowing down to significant ligand-receptor (LR) pairs, we used these to ascertain the molecular subtypes of the samples.

For the clustering process, the “pam” algorithm was employed, and the “Canberra” method was chosen as the distance metric. We executed 500 bootstrap replications, with each cycle including 80% of the patients from the training set. We considered a cluster range from 2 to 10. The most stable clustering solution was pinpointed by examining both the consistency matrix and the consensus cumulative distribution function (CDF), as detailed by Senbabaoglu et al. (26).

### Functional enrichment analysis

To delve into the distinct gene expression landscapes across various molecular subtypes, we implemented Gene Set Enrichment Analysis (GSEA v4.0) (27, 28). We utilized comprehensive gene sets from the Hallmark database (29) and applied specific criteria for statistical significance, including a normalized p-value less than 0.01 and a False Discovery Rate (FDR) below 0.05. For functional characterization of genes that are either upregulated or downregulated, we employed the “clusterProfiler” package (30) to conduct Kyoto Encyclopedia of Genes and Genomes (KEGG) pathway analyses. This helps to identify the biological pathways that are most impacted by the gene expression differences in each subtype.

### Immune cell infiltration analysis

Regarding the immune landscape within the samples, we applied the deconvolution technique known as Cell-type Identification By Estimating Relative Subsets Of RNA Transcripts (CIBERSORT) (31). This enabled us to approximate the proportions of 22 different immune cell types within each sample. Furthermore, we used the ESTIMATE algorithm to assess the relative abundance of immune and stromal cells in the tumor microenvironment.

To evaluate the functionality and presence of T cells within tumors, we utilized the default settings of the TIDE program, which provides scores for T-cell dysfunction and exclusion (32). Additionally, we calculated the single-sample Gene Set Enrichment Analysis (ssGSEA) scores using the GSVA package in R (33), which allowed us to represent the relative enrichment levels of each KEGG pathway. By integrating these multifaceted analyses, we aim to provide a comprehensive overview of the functional roles of genes, the immune cell composition, and their potential implications for patient prognosis and treatment strategies.

### Genomic data exploration and visualization

For our analysis of genomic variations, we sourced the Simple Nucleotide Variation (SNV) dataset from the TCGA, specifically the Level 4 data processed via the “MuTect2” algorithm. This data was retrieved from the Genomic Data Commons (GDC) portal (https://portal.gdc.cancer.gov/).

To examine and illustrate the Single Nucleotide Polymorphisms (SNPs), we utilized the “oncplot” function available in the R package “maftools.” (34), allowing for comprehensive visualization and analysis of these genomic alterations, aiding in the interpretation of their potential biological significance.

### Development of the LR.score prognostic model

To develop a personalized prognostic model, we focused on ligand-receptor (LR) pairs that demonstrated significant relevance for patient outcomes, incorporating them into a penalized Cox regression model using the L1-penalized LASSO (Least Absolute Shrinkage and Selection Operator) methodology, implemented with the R package “glmnet.” The optimal λ value, which controls the strength of the penalty applied to the model, was determined using ten-fold cross-validation, where the data was divided into ten subsets, with each subset used as a validation set once. The optimal λ was chosen based on minimizing the partial likelihood deviance, thereby retaining the most important predictor variables by shrinking the coefficients of less relevant ones to zero, ensuring the best predictive performance while avoiding overfitting. After identifying the predictor variables, we further refined the model using stepwise multivariate regression analysis with the Akaike Information Criterion (AIC) via the “stepAIC” function in the “MASS” package in R. This approach iteratively removed variables to minimize the AIC value, achieving an optimal balance between model complexity and goodness of fit. The final set of 10 LR pairs included in the LR.score were those that remained stable across multiple models and consistently contributed to predicting overall survival. The final risk score for each patient, termed the LR.score, was computed as follows:


$$\text{LR}.\text{score}=\left(\beta_{1}x_{i1}+\;\beta_{2}x_{i2}+\ldots +\;\beta_{p}x_{ip}\right)=\;0.269\;\times \text{ CALCA}\_\text{CALCRL}+0.213\;\times \text{ NCAM}1\_\text{ROBO}3+0.160\;\times \text{ NLGN}1\_\text{NRXN}2\;+\;0.125\times \text{ IL}2\_\text{IL}2\text{RA }-\;0.121\;\times \text{ HGF}\_\text{MET}-\;0.199\;\times \text{ CXCL}12\_\text{ITGA}4\;-\;0.262\;\times \text{ CLEC}11\text{A}\_\text{ITGA}11\;-\;0.317\;\times \text{ AVP}\_\text{AVPR}1\text{B }-\;0.491\;\times \text{ ICAM}4\_\text{ITGA}4\;-\;0.708\;\times \text{ CCL}7\_\text{ACKR}4$$

### Analysis of drug sensitivity in relation to LR.score

To investigate the relationship between drug responsiveness and the LR.score prognostic model, we obtained drug sensitivity data for nearly a thousand cancer cell lines from the Genomics of Drug Sensitivity in Cancer (GDSC) database. We used the Area Under the Curve (AUC) values for various anti-cancer drugs as indicators of drug efficacy. Spearman’s rank correlation analysis was employed to assess the correlation between each drug’s sensitivity and the LR.score. We set a significance threshold at an absolute Spearman’s correlation coefficient (|ρ|) greater than 0.2 and adjusted for False Discovery Rate (FDR) using the Benjamini-Hochberg method with a significance level set at less than 0.05. To further refine drug response predictions, we utilized the “pRRophetic” R package (35). Additionally, for a more targeted approach, we incorporated transcriptomic and clinical data from the IMvigor210 cohort of patients with metastatic bladder cancer who were treated with the anti-PD-L1 drug Atezolizumab. This data was accessed using the “IMvigor210CoreBiologies” R package (36). The immune checkpoints list waw derived from the HisgAtlas database (37).

### Primary AML sample collection and quantitative reverse transcription polymerase chain reaction analysis

Bone marrow specimens were collected from 24 individuals with a primary diagnosis of AML at the Zhuji Affiliated Hospital of Wenzhou Medical University. Additionally, 12 healthy bone marrow samples were donated from individuals undergoing total hip arthroplasty, serving as a control group. All participants provided informed consent, and the study was conducted in line with the Declaration of Helsinki principles. The research was approved from the Ethics Committee of Zhuji People’s Hospital Affiliated to Wenzhou Medical University. Diagnosis for AML was made using the French-American-British (FAB) system, alongside tests such as immunophenotyping, cytogenetic analysis, and molecular genetic profiling. A complete response (CR) to treatment was identified by several criteria, including bone marrow blasts below 5%, no Auer rod-positive blasts, no extramedullary leukemia, an absolute neutrophil count above 1.0 × 10^^9^/L, and a platelet count over 100 × 10^^9^/L. RNA was extracted from various tissues and cells using Trizol reagent as per the manufacturer’s instructions. The reverse transcription step was carried out using the PrimeScript RT reagent Kit. Quantitative real-time PCR (qRT-PCR) was then performed with SYBR Prime Script RT-PCR Kits, following the prescribed procedure. Expression levels of CLEC11A, ICAM4, ITGA4, and AVP were quantified using the 2^-ΔΔCt^ method and normalized against GAPDH mRNA. These expression levels were reported relative to a control level set to a baseline of 1.0. The primer sequences used for amplifying CLEC11A, ICAM4, ITGA4, and AVP genes were as follows: for CLEC11A, forward (5′-GGG CCT CTA CCT CTT CGA AA-3′) and reverse (5′-CAG TTC TCG AGC GTG CCA CC-3′); for ICAM4, forward (5’-GCCTACAGTGAGGGACAGG-3’) and reverse (5’-ATCACGGGCTGCCAGAAG-3’); for ITGA4, forward (5′-TTCCAGAGCCAAATCCAAGAGTAA-3′) and reverse (5′-AAGCCAGCCTTCCACATAACAT-3′); and for AVP, forward (5′-GGGCAGGTAGTTCTCCTCCT-3′) and reverse (5′-CACCTCTGCCTGCTACTTCC-3′).

### Enzyme-linked immunosorbent assay for protein validation

In addition to the mRNA analysis, protein levels of CLEC11A, ICAM4, ITGA4, and AVP were measured using enzyme-linked immunosorbent assays (ELISAs). Bone marrow plasma was separated by centrifugation at 1500 × g for 10 minutes at 4°C from the same 24 AML patients and 12 healthy controls as described above and stored at -80°C until analysis. ELISA kits specific for human CLEC11A (#ELK6393), ICAM4 (#ELK3048), ITGA4 (#ELK9691), and AVP (#ELK5414) were used according to the manufacturer’s protocols. All reagents and samples were brought to room temperature (18-25°C) before use. The 25× Wash Buffer was diluted to 1× with double-distilled water. Standard dilutions were prepared from a stock concentration of 60 ng/mL to create a standard curve with points at 60, 30, 15, 7.5, 3.75, 1.88, and 0.94 ng/mL. 100 μL of each standard, control, or sample was added in duplicate to the appropriate wells of a pre-coated microplate, which was then incubated at 37°C for 80 minutes. After incubation, the wells were washed three times with 200 μL of 1× Wash Buffer, followed by the addition of 100 μL of Biotinylated Antibody Working Solution to each well. The plate was incubated for another 50 minutes at 37°C, and the washing step was repeated. Next, 100 μL of Streptavidin-HRP Working Solution was added to each well, followed by incubation at 37°C for 50 minutes and another washing step repeated five times. The colorimetric reaction was developed by adding 90 μL of TMB Substrate Solution to each well, incubating the plate in the dark at 37°C for 20 minutes. The reaction was stopped by adding 50 μL of Stop Solution, changing the color from blue to yellow. Absorbance was immediately measured at 450 nm using a microplate reader (R&D, MN, USA). The concentration of each protein in the samples was then determined by comparing the corrected absorbance values to the standard curve generated from the known concentrations of the standards.

### Statistics

For the evaluation of statistical differences between two continuous and normally distributed variables, we utilized the unpaired Student’s t-test. In cases where the variables were not normally distributed, the Wilcoxon rank-sum test was employed instead. For comparisons involving three or more groups with non-parametric distributions, Kruskal-Wallis tests were conducted. For examining relationships between categorical variables, Fisher’s exact test was implemented. Correlation analyses were conducted using Spearman’s rank correlation test to assess the strength and direction of associations between variables. All visualizations and statistical computations were carried out using R software, version 4.3.2, provided by the R Foundation for Statistical Computing in Vienna, Austria.

## Results

### Identification and analysis of prognostic LR pairs in acute myeloid leukemia

To pinpoint Ligand-Receptor (LR) pairs significantly correlated with the survival outcomes in AML, we incorporated data from three distinct AML cohorts: TCGA-LAML, GSE37642, and GSE12417. Initially, survival analysis for these LR pairs was executed separately for each cohort. This was followed by a meta-analysis where we amalgamated the P-values pertaining to the prognostic importance of these LR pairs from all three cohorts (adjust P-value< 0.01). Upon completing multiple hypothesis testing corrections, we isolated 94 LR pairs that demonstrated substantial prognostic relevance. Of these, 56 LR pairs were indicative of a poor prognosis, while 38 suggested a favorable outcome (**Figure 1B**; **Supplementary Table S2**). The interactive network involving these LR pairs is depicted in (**Figure 1C**).

Additionally, we undertook KEGG pathway enrichment analysis targeting the specific ligands and receptors from these pairs. Remarkably, these 94 LR pairs were predominantly enriched in key biological pathways. These include Cytokine−cytokine receptor interaction, Viral protein interaction with cytokine and cytokine receptor, PI3K−Akt signaling pathway, Proteoglycans in cancer, Cell adhesion molecules (CAMs), and ECM−receptor interaction (**Figure 1D**).

### Molecular classification of ligand-receptor pairs

**Figure 2:**
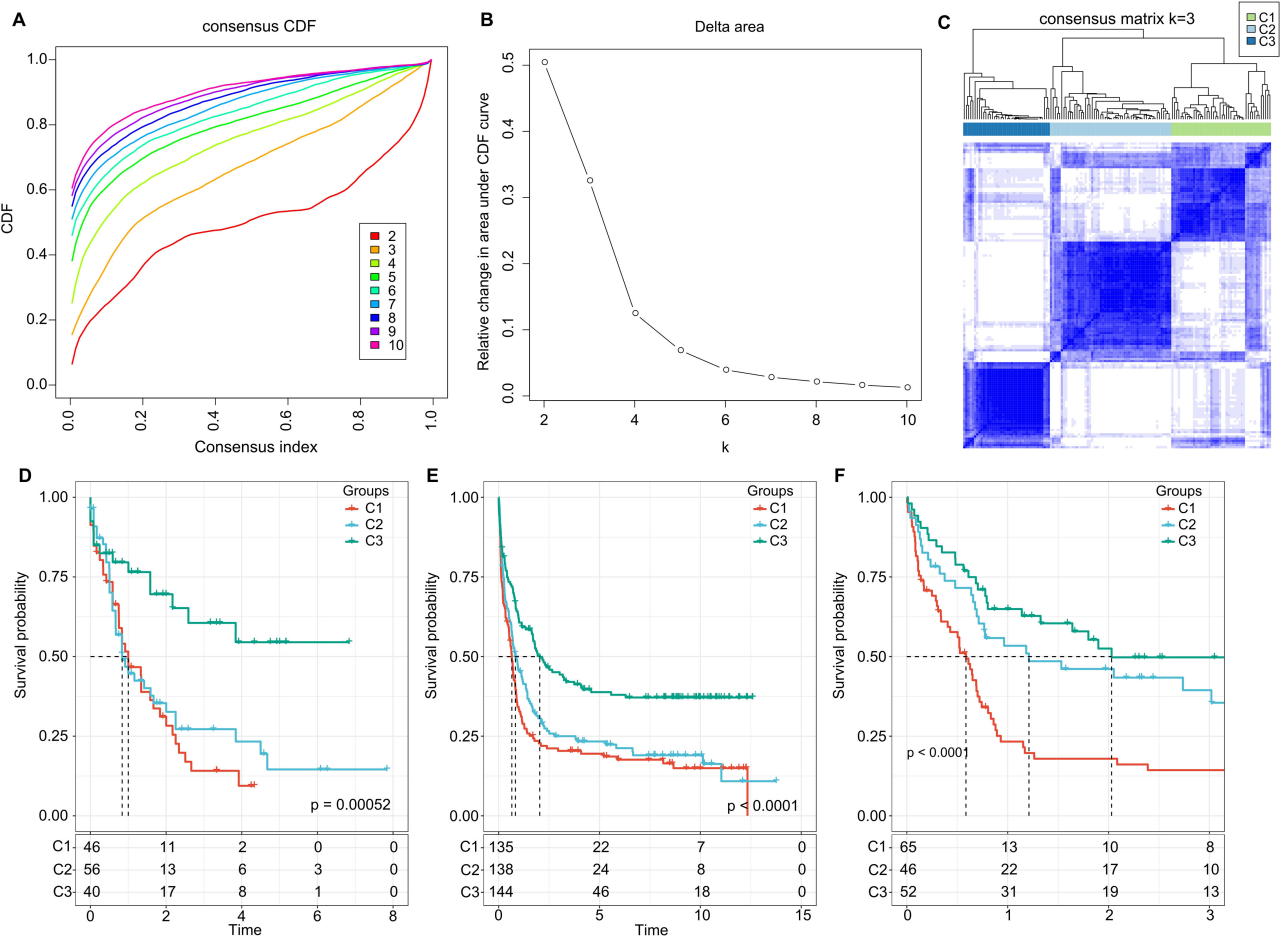
Cluster Analysis and Survival Outcomes Based on LR Pairs. **(A)** Consensus clustering using LR pairs and CDF curves from the consensus clustering in the TCGA dataset. **(B)** Delta area plot from the consensus clustering in the TCGA dataset. **(C)** Consensus matrices showcasing the identified clusters (k = 3). **(D)** Overall survival Kaplan-Meier plot for the three subtypes in the TCGA cohort (P = 0.00052, log-rank test). **(E)** Overall survival KaplanMeier plot for the three subtypes in the GSE37642 dataset (P< 0.0001, log-rank test). **(F)** Overall survival Kaplan-Meier plot for the three subtypes in the GSE12417 dataset (P< 0.0001, log-rank test).

Further, we summed the expression levels of the receptor and ligand genes to represent the expression intensity of each LR pair. We then used the gene expression levels of these LR pairs for molecular subtyping. In this step, we included the 94 LR pairs that were identified in the previous analysis as being significantly correlated and prognostically relevant. Using Consensus Clustering, we clustered 142 AML samples from the TCGA cohort. Based on the Cumulative Distribution Function (CDF), we determined the optimal number of clusters. The CDF Delta area curve suggested that choosing three clusters provided the most stable clustering results (as shown in **Figures 2A, B**). Ultimately, we selected k=3 to obtain three molecular subtypes (**Figure 2C**). Upon further analysis of the prognostic features of these three subtypes, we observed significant prognostic differences among them. Specifically, subtype C3 showed better prognosis, while subtype C1 had poorer outcomes (*P* = 0.00052; **Figure 2D**). Additionally, using the same methodology, we conducted molecular subtyping for the AML patient cohort in GSE37642, and observed similar significant prognostic differences among these three subtypes (*P*< 0.0001; **Figure 2E**), consistent with the training set. The same phenomenon was also observed in the GSE12417 cohort (*P*< 0.0001; **Figure 2F**).

**Figure 3:**
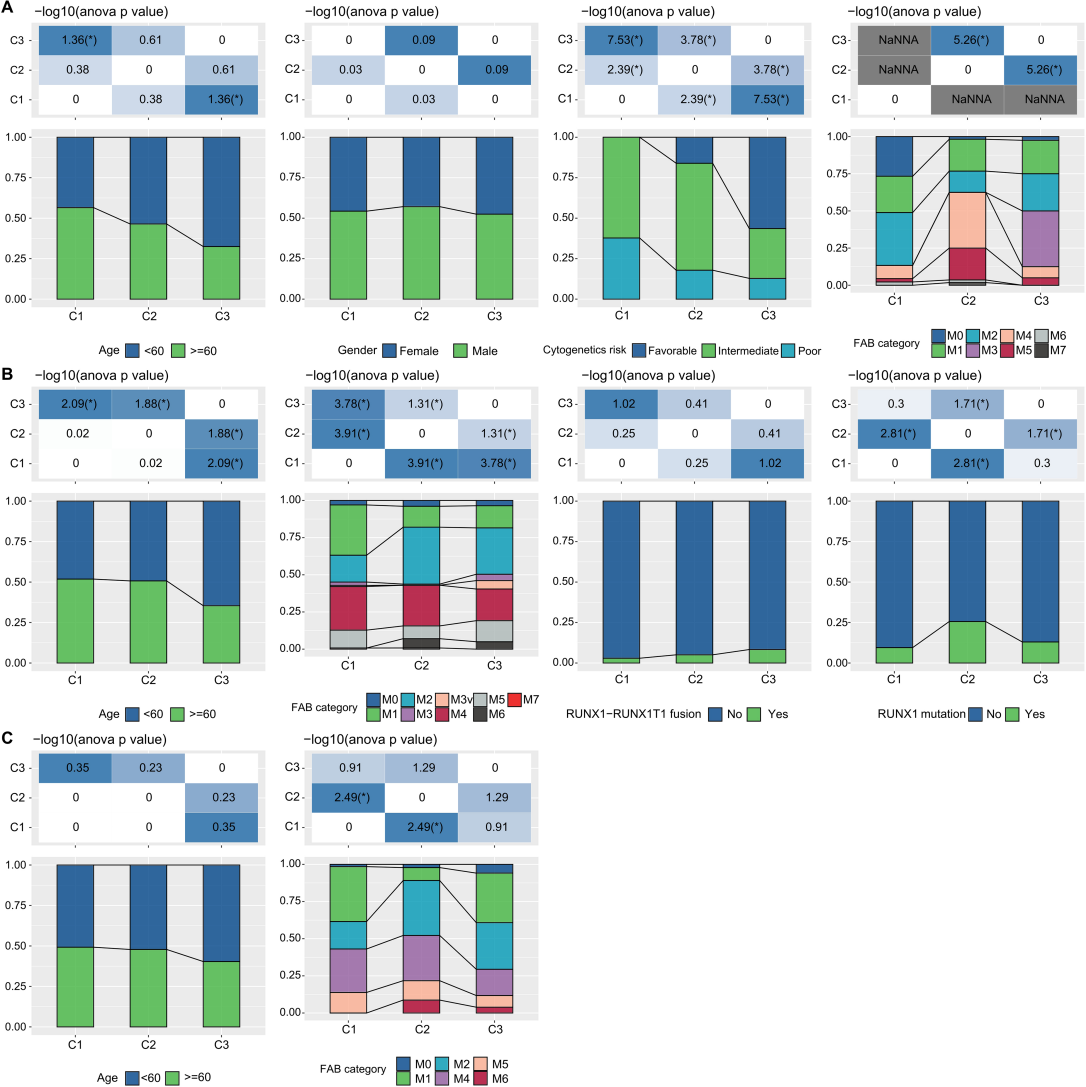
LR pairs-based clusters and clinical characteristics **(A)**. Distribution of clinical information for molecular subtypes in the TCGA cohort (Fisher’s exact test). *P< 0.05. **(B)** Distribution of clinical information for molecular subtypes in the GSE37642 cohort (Fisher’s exact test). *P< 0.05. **(C)** Distribution of clinical information for molecular subtypes in the GSE12417 cohort (Fisher’s exact test). *P< 0.05.

Additionally, in the TCGA dataset, we compared the distribution of various clinical pathological features across the three molecular subtypes to examine if these features varied among the subtypes. We found that there were differences in “age” groups, “CALGB Cytogenetics Risk Category,” and “FAB Category” among the three molecular subtypes. Specifically, the C1 subtype had a significantly higher proportion of older patients compared to the C3 subtype (Fisher’s exact test, -log10 P-value = 4.74). Most patients in the C3 subtype fell into the “Favorable” (Fisher’s exact test, -log10 P-value = 7.53) (**Figure 3A**). Similarly, we examined differences in clinical information among different molecular subtypes in the GSE37642 and GSE12417 cohorts (**Figures 3B, C**). We observed that the majority of patients did not have RUNX1 mutations. These analyses further support the notion that these molecular subtypes not only have prognostic value but also correlate with various clinical and pathological features, thereby potentially aiding in more targeted treatment approaches.

### Mutation characteristics across different molecular subtypes

**Figure 4:**
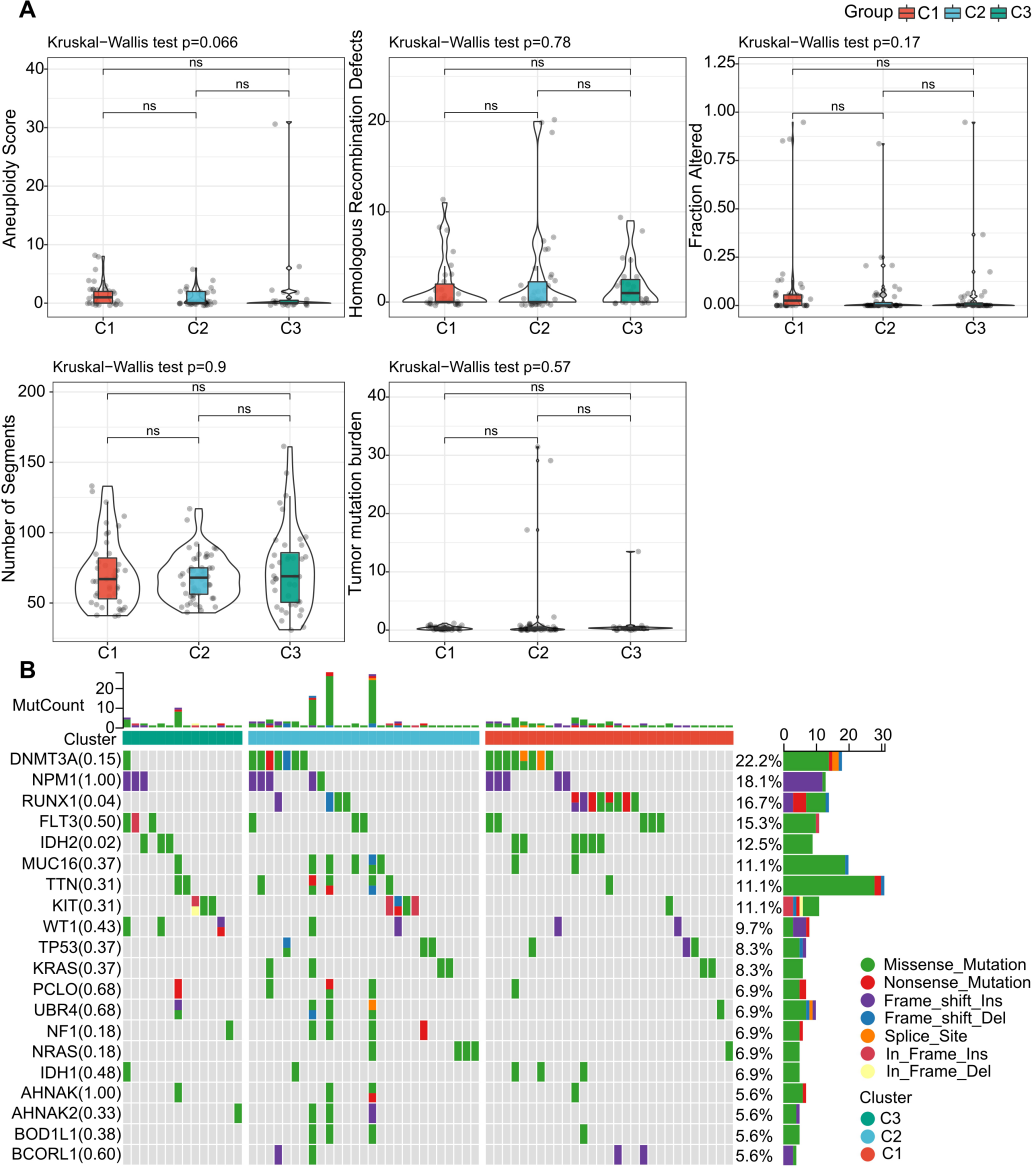
Genomic Variation Patterns Across LR Pair-Defined Clusters. **(A)** Analysis of parameters such as aneuploidy score, homologous recombination deficits, segment count, fraction of alterations, and tumor mutation burden within the LR pair-based clusters. Significance levels: ns, P > 0.05; *P < 0.05; **P < 0.01; ***P < 0.001. Kruskal-Wallis test employed. **(B)** Mutation profile showcasing the top 20 prevalent mutations across 142 AML patients. The top panel provides a count of mutations for each individual, whereas the bar chart below denotes the LR pair-defined clusters. An accompanying legend depicts various genetic mutation types and their occurrences.

We further investigated the differences in genomic alterations among the three molecular subtypes within the TCGA cohort. Our analysis revealed that there were no significant differences among the molecular subtypes in terms of Aneuploidy Score, Homologous Recombination Defects, Fraction Altered, Number of Segments, and Tumor Mutation Burden (**Figure 4A**).

Moreover, we scrutinized the variations in gene mutations across the distinct molecular subtypes. We highlighted the top 20 genes that showed substantial differences in their mutation rates (**Figure 4B**). Of particular interest were genes like DNMT3A, NPM1, and RUNX1, which exhibited noticeable disparities in mutation frequency among the three molecular subgroups. Intriguingly, a higher prevalence of RUNX1 and DNMT3A mutations was observed in subtypes C1 and C2, and these mutations were correlated with unfavorable prognostic outcomes. This observation aligns with existing scientific literature that associates these specific mutations with poorer survival rates in AML (38, 39).

These findings imply that while some genomic features like Aneuploidy Score and Tumor Mutation Burden may not differ significantly among the subtypes, specific genes do show variations in mutation rates. This could have implications for understanding the biological distinctions among the subtypes and potentially for targeted therapeutic strategies.

### Functional annotation of ligand-receptor pairs-based clustering

**Figure 5:**
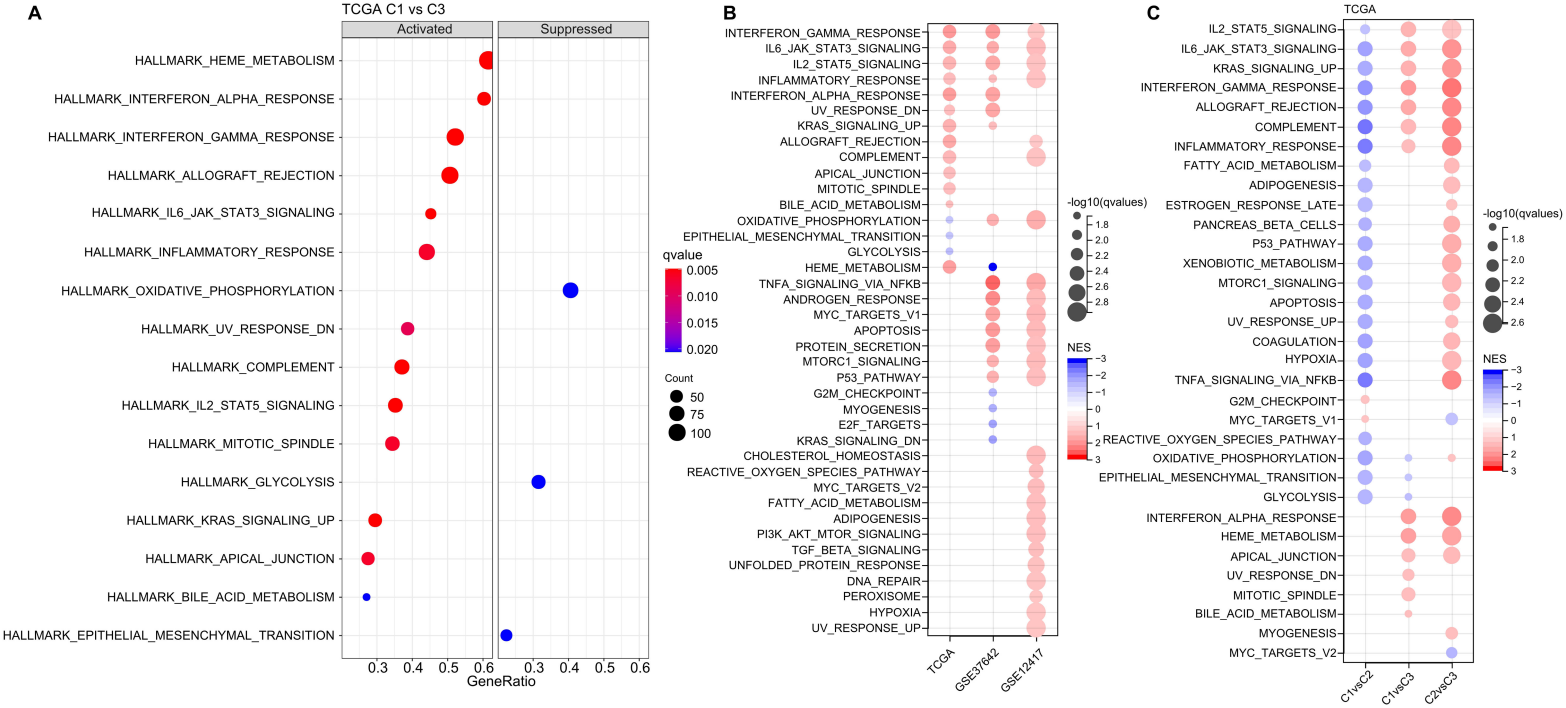
Analysis of Pathway Enrichment in LR-Based Clusters. **(A)** GSEA output contrasting C1 vs C3 in the TCGA-LAML dataset. **(B)** Visual representation of GSEA findings when comparing C1 vs C3 subtypes across three AML cohorts: TCGA-LAML, GSE37642, and GSE12417. **(C)** A color-coded bubble chart detailing GSEA results for various molecular subtypes within the TCGA-LAML dataset. Blue bubbles signify downregulated hallmarks, while red bubbles point to upregulated ones.

Next, we investigated whether there were differentially activated pathways within the various molecular subgroups. To identify these pathways, we performed Gene Set Enrichment Analysis (GSEA) using all candidate gene sets from the Hallmark database, with FDR threshold less than 0.05 for significant enrichment. In the TCGA cohort, when compared to the C3 subtype, the C1 subtype exhibited activation of 13 pathways and suppression of 3 pathways, as shown in (**Figure 5A**). These pathways were mainly related to immune responses, such as INTERFERON_GAMMA_RESPONSE, INFLAMMATORY_RESPONSE, INTERFERON_ALPHA_RESPONSE, ALLOGRAFT_REJECTION, and COMPLEMENT. In addition, we analyzed the significant enriched gene sets in the two validation cohorts when comparing the C1 subtype with the C3 subtype. Generally, activated pathways primarily included immune markers, such as INTERFERON_GAMMA_RESPONSE, IL6_JAK_STAT3_SIGNALING, IL2_STAT5_SIGNALING, and INFLAMMATORY_RESPONSE (**Figure 5B**). Furthermore, we compared differential pathways between C1 and C2, C1 and C3, as well as C2 and C3 in TCGA cohort. As shown in (**Figure 5C**),

we identified a notable concentration of immune-related pathways among the diverse subtypes. GSEA analysis across these subtypes revealed that individuals in the C1 subtype typically displayed heightened activity in immune regulatory pathways. This leads us to hypothesize that the ligand-receptor pairs utilized for molecular classification might have significant influence in shaping the immune microenvironment.

**Figure 6:**
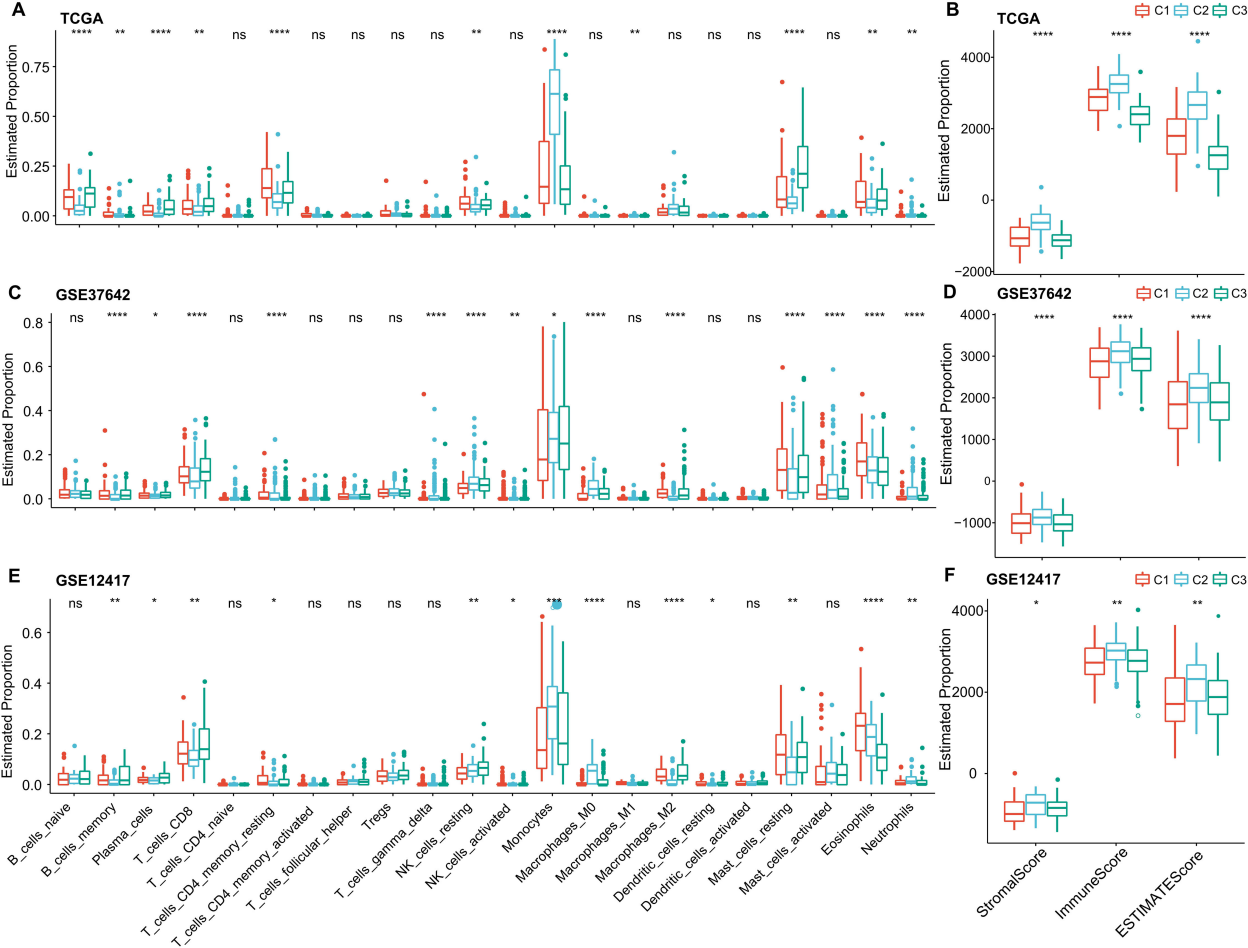
Immune Cell Variability Across Different Molecular Subtypes in Three AML Cohorts. **(A–F)** Differences in immune cell infiltration among subtypes, as determined by CIBERSORT and ESTIMATE. Significance levels: ns, P > 0.05; *P > 0.05; **P > 0.01; ***P > 0.001; ****P > 0.0001. Analyzed using the Kruskal-Wallis test.

To delve deeper into the variations in the immune microenvironment across patients from different molecular groups, we assessed the relative proportions of 22 immune cell types across the three AML cohorts using the CIBERSORT algorithm. This revealed pronounced differences in the presence of specific immune cells across the three molecular subtypes (as illustrated in (**Figures 6A, C, E**). Notably, the C3 subtype exhibited elevated levels of CD8 T cells and plasma cells, suggesting heightened adaptive immune responses. Additionally, we employed the ESTIMATE algorithm to assess immune cell infiltration, as shown in (**Figures 6B, D, F**). We found that the “ImmuneScore” was consistently highest in the C2 subtype across the TCGA, GSE37642, and GSE12417 cohorts, indicating a relatively higher level of immune cell infiltration in the C2 subtype, importantly, C2 also has highest myeloid lineage cells compare to C1 and C2.

### Development of a scoring model based on ligand-receptor pairs

We found that molecular subtypes based on LR pairs exhibit different mutation landscapes, distinct pathway features, and varying levels of immune infiltration. To further refine our risk model, we employed lasso regression on the 94 significant LR pairs identified from the meta-analysis within the TCGA-LAML cohort. We showed that as the value of lambda increases, the number of coefficients tending towards zero also increases (**Supplementary Figure S1A**). Using 10-fold cross-validation, we identified an optimal lambda value of 0.0868 and selected 13 LR pairs for further analysis (**Supplementary Figure S1B**). Subsequently, we used stepwise multivariate regression analysis and employed the Akaike Information Criterion (AIC) to further refine our model, ultimately identifying 10 key LR pairs. These pairs include “AVP->AVPR1B,” “CALCA->CALCRL,” “CCL7->ACKR4,” “CLEC11A->ITGA11,” “CXCL12->ITGA4,” “HGF->MET,” “ICAM4->ITGA4,” “IL2->IL2RA,” “NCAM1->ROBO3,” and “NLGN1->NRXN2” (**Supplmentary Figure S1C**).

**Figure 7:**
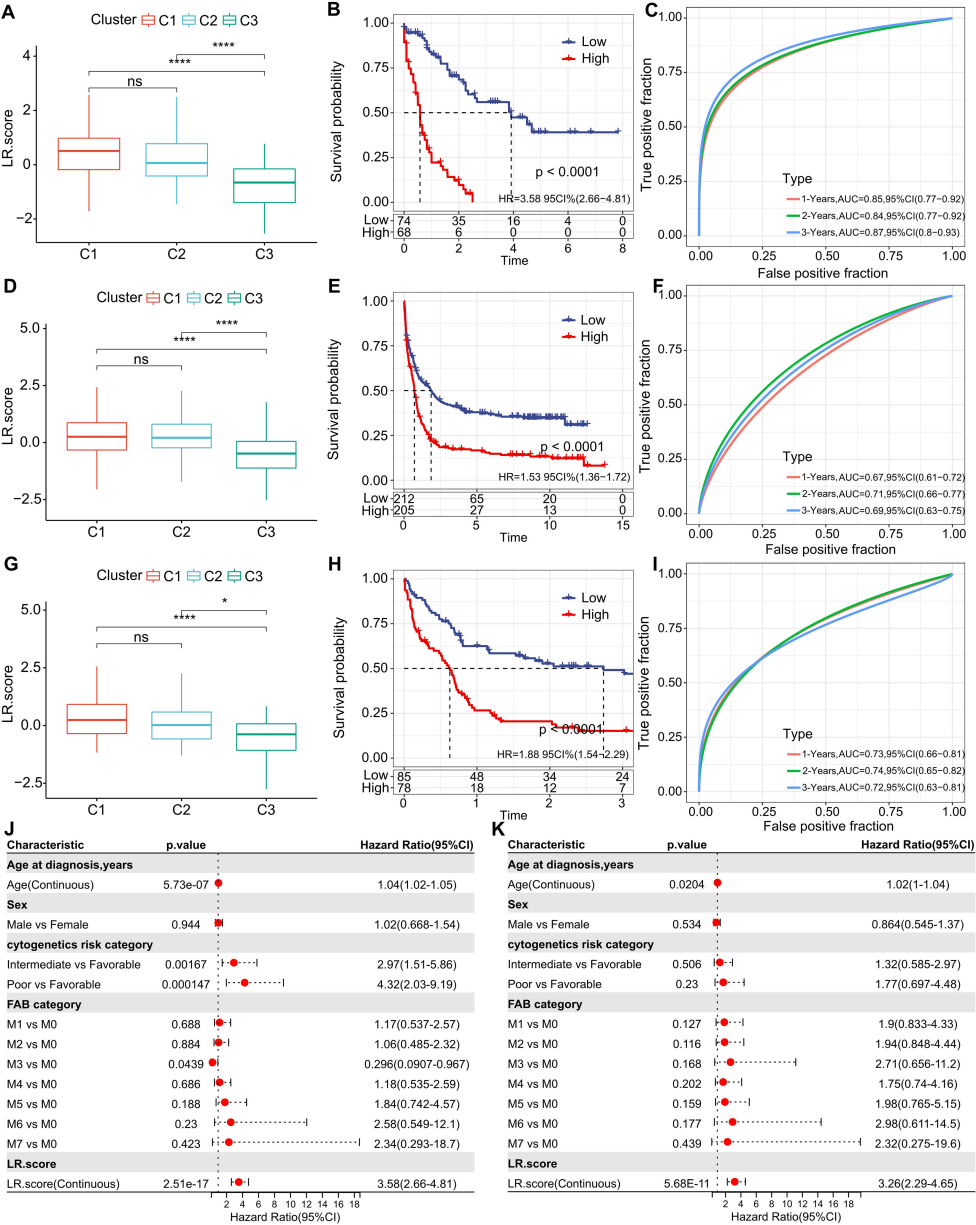
Understanding the LR.score Across Cohorts. **(A)** Variability in LR.score among the TCGA-LAML cohort. **(B)** Survival comparison between high and low LR.score groups in TCGA-LAML. **(C)** Predictive accuracy of LR.score in the TCGA-LAML cohort for 1, 2, and 3-year survival. **(D-I)** Similar analyses conducted for GSE37642 and GSE12417 cohorts. **(J, K)** Univariate and multivariate Cox regression model analyses factoring in LR.score, age, gender, cytogenetics risk, FAB category, and outcomes in the TCGA-LAML cohort.

Building on the 10 distinguished ligand-receptor (LR) pairs, we developed an LR scoring system, termed LR.score, to quantifiably gauge the activity patterns of these LR pairs in AML patients. We observed a notably elevated LR.score in patients of the “C1” subtype in comparison to their “C3” counterparts (**Figure 7A**). Probing the clinical relevance of the LR.score, we categorized patients into high and low LR.score groups, using “0” as the demarcation point. Interestingly, those with a diminished LR.score exhibited a pronounced survival advantage (**Figure 7B**; log-rank test, P< 0.0001). The receiver operating characteristic (ROC) curve’s area under the curve (AUC) for 1-, 3-, and 5-year overall survival stood at 0.85, 0.84, and 0.87, respectively (**Figure 7C**). In our validation datasets, GSE37642 and GSE12417, a similar pattern emerged where the LR.score for “C1” was appreciably higher than for “C2” and “C3” (Wilcoxon rank-sum test, P< 0.001) (**Figures 7D, G**). In alignment with our initial observations, patients with a reduced LR.score in these validation sets also showcased a marked survival benefit (**Figures 7E, H**; log-rank test, P< 0.0001). The AUC values from the ROC assessments were 0.67, 0.71, and 0.69 for 1-, 3-, and 5-year overall survival in GSE37642, and 0.73, 0.74, and 0.72 in GSE12417, respectively. The consistency in AUC values across these intervals underscores the reliability of the model’s prognostic potential (**Figures 7F, I**).

To examine whether the LR.score could serve as an independent prognostic factor, we performed both univariate and multivariate Cox regression analyses using patient clinical features such as age, gender, cytogenetics risk category, and FAB category. We found that the LR.score is a reliable and independent prognostic biomarker (**Figures 7J, K**; HR=3.26, 95% CI 2.29-4.65, *P* = 5.68E-11).These results suggest that the LR.score can reflect the LR-pairs patterns in AML patients and predict prognosis.

**Figure 8:**
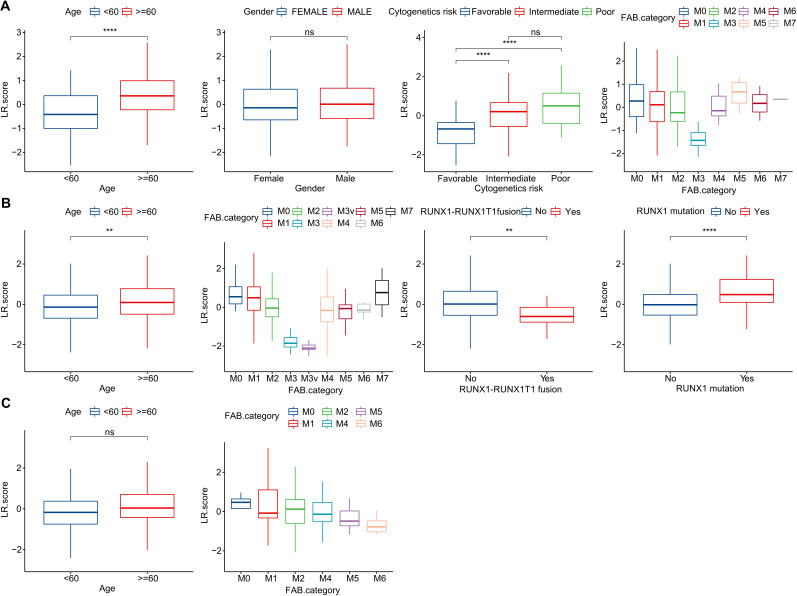
Clinical Implications of the LR.score. **(A–C)** Distribution of LR.score concerning various clinical-pathological features across TCGA-LAML, GSE37642, and GSE12417 cohorts. Significance denoted as: ns, *P* > 0.05; ** *P* < 0.01; ****P < 0.0001. Analyzed using the Wilcoxon rank-sum test.

To investigate the relationship between the LR.score and clinical characteristics of AML, we analyzed the differences in the LR.score based on various clinical-pathological features in the TCG dataset. Our findings indicated that as age increases, the LR.score also rises. Moreover, higher “cytogenetics risk” levels were associated with elevated LR.score values (**Figure 8A**). We further compared the relationship between patients’ LR.score and their clinical-pathological characteristics in the GSE37642 and GSE12417 cohorts (**Figures 8B, C**). It was observed that older patients tended to have higher LR.score as well as RUNX1 mutation patients. In summary, the higher the clinical staging level of the patient, the greater the LR.score.

### LR.score and relevant biological functions

**Figure 9:**
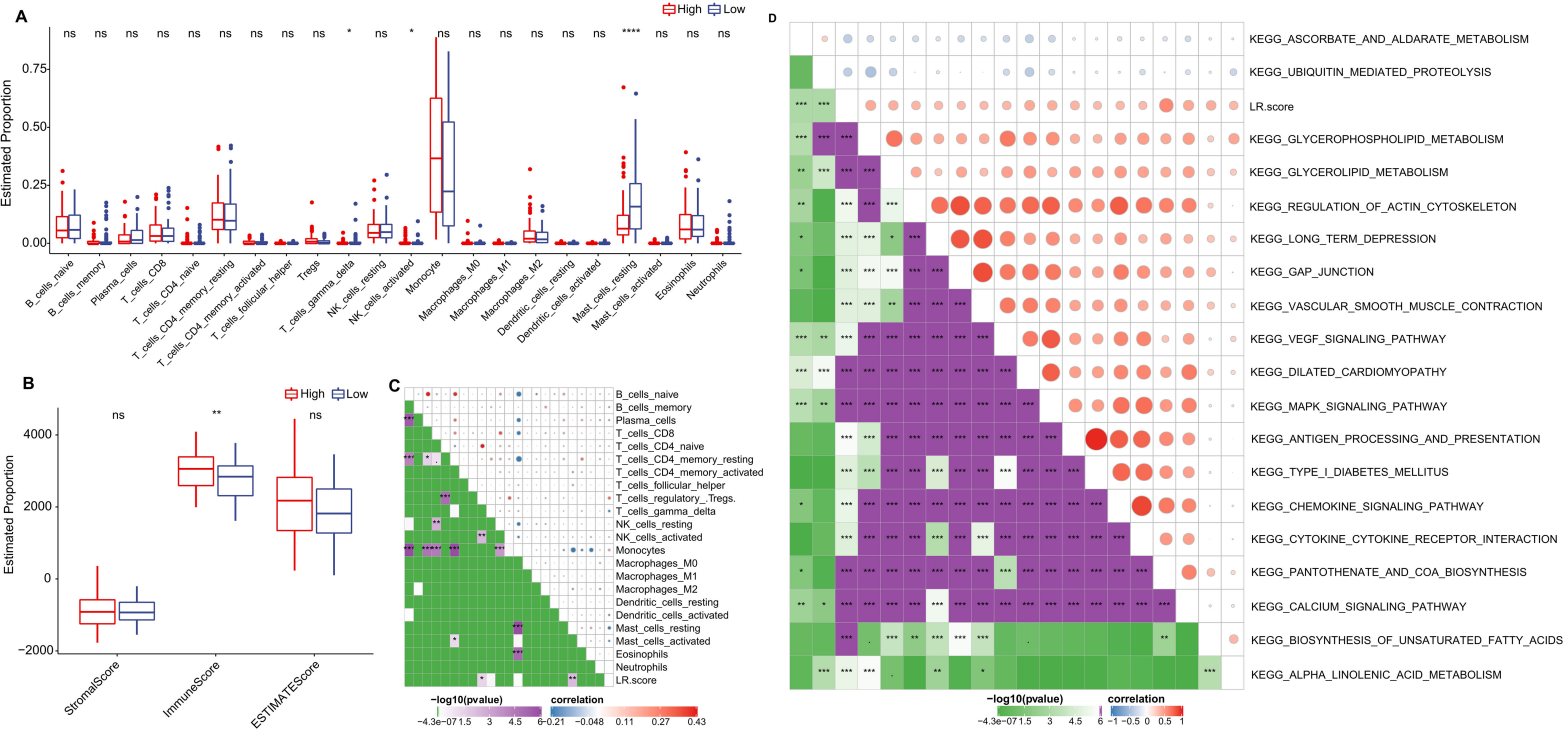
Role of LR.score in Immune Infiltration and Pathway Activation. **(A, B)** Distribution of immune cell types and stromal-immune scores between high and low LR.score groups in the TCGA-LAML cohort. **(C, D)** Correlation analyses between immune cell components, KEGG pathways, and the LR.score.

We further delved into the distribution differences of scores for 22 immune cell types across LR.score groupings in the TCGA cohort, as depicted in (**Figure 9A**). Generally, scores for most immune cells do not display significant disparities between LR.score groups. However, certain cells like T_cells_gamma_delta, NK_cells_activated, and Mast_cells_resting exhibited notable differences. Moreover, when comparing immune infiltration, the ImmuneScore was consistently lower in the low LR.score group than in the high LR.score group (**Figure 9B** **).** Additionally, Employing Pearson’s correlation coefficient, we evaluated the association between the LR.score and immune cell infiltration, the results of which are shown in (**Figure 9C**). Interestingly, a strong positive correlation was observed between LR.score and T_cells_regulatory (Tregs), while a significant negative correlation was noted with Mast_cells_resting.

To understand the relationship between LR.score and biological functionality, we employed single-sample GSEA analysis (ssGSEA) on the gene expression profiles of AML samples from the TCGA cohort. This enabled us to compute scores for each sample across various functionalities, leading to ssGSEA scores for each function across individual samples. Further correlation analysis between these functional scores and LR.score revealed functions with correlations greater than 0.35, as shown in (**Figure 9D**) and (**Supplementary Table S3**). As a result, 17 pathways positively correlated with the LR.score of samples, while 2 pathways demonstrated a negative correlation. Notably, cancer-related pathways such as KEGG_GLYCEROPHOSPHOLIPID_METABOLISM, KEGG_GLYCEROLIPID_METABOLISM, KEGG_REGULATION_OF_ACTIN_CYTOSKELETON, and KEGG_MAPK_SIGNALING_PATHWAY were positively correlated with LR.score.

### LR.score model in immunotherapy/chemotherapy

**Figure 10:**
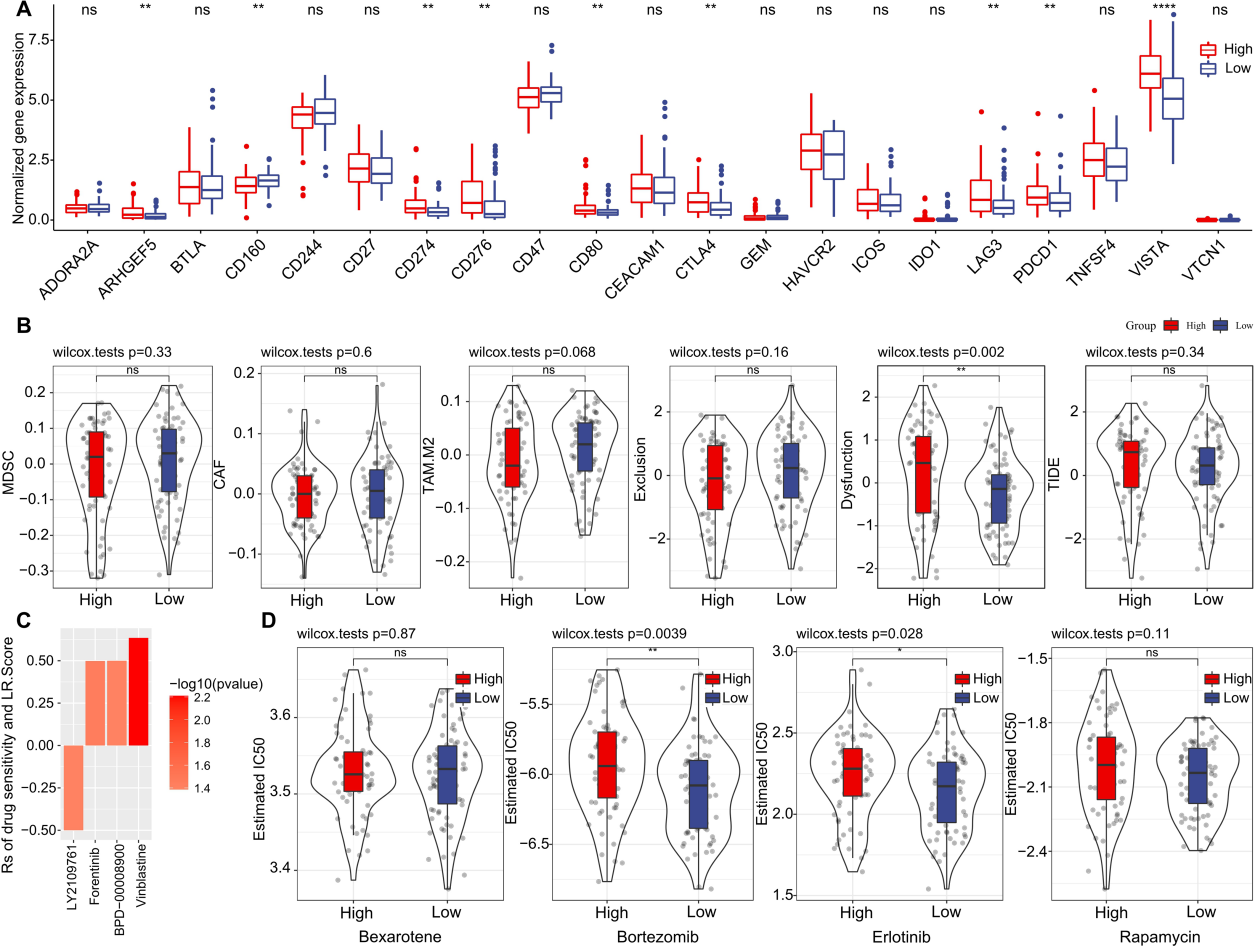
Drug Sensitivity Predictions Using the LR.score. **(A)** Immune checkpoint expression differences between LR.score groups in TCGA-LAML. **(B)** Comparing scores related to immune activity between high and low LR.score groups. **(C)** Correlation between drug sensitivity and the LR.score, including the Estimated IC50 values for various drugs. **(D)** Comparing scores related to chemotherapy drugs between high and low LR.score groups. ns, *P* > 0.05; *P < 0.05; **P < 0.01; ****P < 0.0001.

Furthermore, we investigated if there were disparities between LR.score groupings concerning their responses to treatment. Initially, we compared the expression of immune checkpoints between the LR.score groups. As shown in (**Figure 10A**), certain immune checkpoint genes exhibited differential expression across the LR.score groups. Specifically, high gene expressions of ARHGEF5, CD274, CD80, CTLA4, LAG3, PDCD1, and VISTA are associated with high LR.score (*P*< 0.05, Wilcoxon rank-sum test).

We subsequently examined the disparities in immunotherapy outcomes between the LR.score categories. Utilizing the TIDE algorithm, we gauged the probable clinical ramifications of immunotherapy among our designated high and low LR.score groups. Notably, a heightened TIDE prediction score implies an augmented propensity for immune evasion, hinting at potentially diminished benefits from immunotherapy for the patient. As illustrated in (**Figure 10B**) and **(Supplementary Table S4**), discernible distinctions between high- and low- LR.scores in MDSC, CAF, and TIDE scores were absent. Simultaneously, we contrasted the anticipated T-cell dysfunction and exclusion scores across various metabolic molecular subtypes within the TCGA cohort. The group with elevated LR.score exhibited the most pronounced T-cell dysfunction score (P< 0.01, Wilcoxon rank-sum test).

Delving deeper into the ramifications of LR.score on drug responsiveness, we explored its association with reactions in tumor cell lines to drugs. Through Spearman correlation analysis, we discerned four notable associations between LR.score and drug susceptibility in the Genomics of Drug Sensitivity in Cancer (GDSC) database (**Figure 10C**). Among these, three correlations indicated drug resistance in tandem with the LR.score, encompassing Forentinib, BPD-00008900, and Vinblastine. We also gauged the variances in responses to prevalent chemotherapy agents such as ‘Bexarotene’, ‘Bortezomib’, ‘Erlotinib’, and ‘Rapamycin’ between the LR.score categories (**Figure 10D**). Observations revealed that patients with a diminished LR.score manifested heightened sensitivity to drugs, notably Bortezomib (*P* = 0.0039, Wilcoxon rank-sum test) and Erlotinib (*P* = 0.028, Wilcoxon rank-sum test), as compared to their high LR.score counterparts. Collectively, these insights underscore a linkage between LR pairs and drug sensitivity, positing the LR.score as a potential biomarker to inform tailored therapeutic approaches.

### Validation of the LR.score model genes

**Figure 11:**
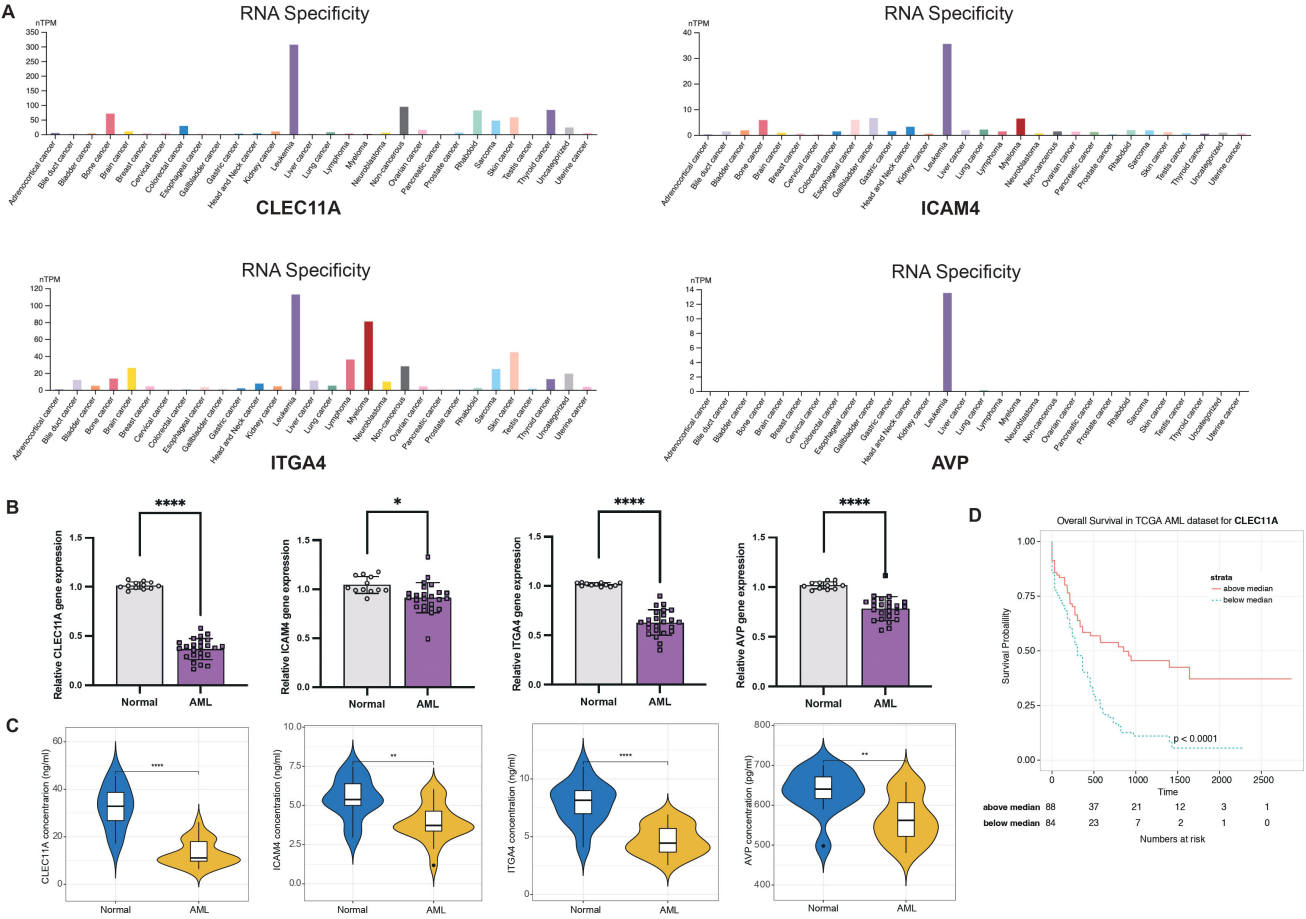
LR.score model validation. **(A)** Cancer specificity of CLEC11A, ICAM4, ITGA4, and AVP expressions among pan-cancer. **(B)** qRT-PCR of CLEC11A, ICAM4, ITGA4, and AVP comparing normal and AML bone marrows. Significance denoted as: *P< 0.05; ****P< 0.0001. Analyzed using the unpaired t test. **(C)** ELISA concentration of CLEC11A, ICAM4, ITGA4, and AVP comparing normal and AML bone marrows. Significance denoted as: **0.001< *P*< 0.01; ****P< 0.0001. Analyzed using the unpaired t test. **(D)** Overall survival Kaplan-Meier plot for high- and low- CLEC11A expressions in the TCGA dataset (*P*< 0.0001, log-rank test).

Considering the LR.score model’s proven capability in predicting the risk and treatment outcomes for AML, we aim to validate the individual gene expression profiles to determine whether the model possesses the requisite sensitivity and specificity to be applicable for validation within our patient cohort. First of all, we investigated the cancer specificity of certain gene-protein pairs by checking the cell line database in The Human Protein Atlas (HPA), focusing on the following associations: “AVP->AVPR1B,” “CALCA->CALCRL,” “CCL7->ACKR4,” “CLEC11A->ITGA11,” “CXCL12->ITGA4,” “HGF->MET,” “ICAM4->ITGA4,” “IL2->IL2RA,” “NCAM1->ROBO3,” and “NLGN1->NRXN2”. Our analysis revealed that, among the genes studied, CLEC11A, ICAM4, ITGA4, and AVP exhibited the highest specificity to AML when compared to other cancer types. This suggests a strong association between the expression levels of these genes and AML, pointing to their potential role as specific biomarkers for this disease (**Figure 11A**). Next, we examined the expression of CLEC11A, ICAM4, ITGA4, and AVP in 12 normal and 24 AML bone marrow samples by quantitative reverse-transcription polymerase chain reaction (qRT-PCR). In comparison to the healthy bone marrow (**Figure 11B**), the levels of CLEC11A, ITGA4, and AVP were notably reduced in samples from AML patients, with a highly significant statistical difference (*P*< 0.0001). The expression of ICAM4 was also found to be slightly diminished (*P*< 0.05). These findings indicate that CLEC11A, ICAM4, ITGA4, and AVP may act as inverse biomarkers in the context of AML. Additionally, we used enzyme-linked immunosorbent assay (ELISA) to further validation these genes in the protein level. As shown in **Figure 11C**, the protein concentrations of CLEC11A and ITGA4 were significantly higher in normal samples compared to AML (*P*< 0.0001), and the same trend was observed for ICAM4 and AVP (*P*< 0.01). Furthermore, survival analysis reveals that patients with higher levels of CLEC11A has a better prognosis than those with lower expression (**Figure 11D**, *P*< 0.0001), reinforcing the potential role of CLEC11A as a particularly significant negative biomarker.

## Discussion

In our research, we pinpointed 94 prognostic LR pairs that predominantly participate in pathways such as Cytokine-cytokine receptor interactions, PI3K-Akt signaling, and Proteoglycans in cancer. This indicates that specific LR pairs significantly influence AML survival by modulating key oncogenic signaling pathways. Based on these survival-related LR pairs, we delineated three distinct molecular subtypes. Furthermore, we devised a prognostic scoring model anchored on 10 LR pairs, and its predictive efficacy was corroborated in separate cohorts. The LR.score model offers a novel advantage over existing AML prognostic models by focusing on ligand-receptor interactions within the tumor microenvironment, providing insights into the dynamic cell-cell communication that drives disease progression and therapeutic response. This model not only enhances prognostic accuracy by identifying distinct molecular subtypes but also predicts responses to targeted therapies, thereby facilitating more personalized treatment strategies. Notably, this scoring system provided insights into chemotherapy responsiveness and probable outcomes to immune checkpoint blockade treatments in AML patients. Our findings underscore the potential of LR pair-driven gene signatures as prospective biomarkers for both prognosis and therapeutic response prediction in AML.

The C1 subtype was distinguished by its association with the worst prognosis, also demonstrated a notable immune cell infiltration, as evidenced by the higher immune score. The C3 subtype was marked by a more favorable prognosis when compared to the other subtypes. Additionally, patients within the C3 subtype demonstrated higher CD8 T cell and plasma cell levels, indicative of enhanced adaptive immune activities. The C3 subtype, with its lower LR.score, showed potential sensitivity to ICB treatments. This aligns with recent research that has indicated a synergistic therapeutic potential when combining targeted therapies with immunotherapies (40–45).

In our analysis of genomic alterations across the three molecular subtypes within the TCGA cohort, we observed no significant differences in traditional markers of genomic instability, such as Aneuploidy Score, Homologous Recombination Defects, Fraction Altered, Number of Segments, and Tumor Mutation Burden. These predominantly negative results suggest that these conventional genomic features are not the primary drivers of the distinct molecular and clinical characteristics seen in each AML subtype (7, 46). This finding underscores the complexity of AML and highlights the potential importance of other factors, such as epigenetic modifications, microenvironmental influences, or specific signaling pathways activated by ligand-receptor (LR) pairs, in defining the disease behavior and therapeutic response. The lack of significant differences in these traditional genomic metrics reinforces the need to explore alternative mechanisms beyond standard genomic alterations, emphasizing the critical role of LR interactions in understanding AML heterogeneity and in identifying novel therapeutic targets.

The roles of specific mutations, particularly DNMT3A, RUNX1, NPM1C, and Flt3, in the progression and prognosis of AML have been the focus of several studies. DNMT3A mutations, often found in about a third of AML patients, have been associated with adverse outcomes and are often linked with a more aggressive disease course and shorter overall survival rates (38, 47, 48). Similarly, RUNX1 mutations are known to be recurrent in AML and have been associated with lower complete remission rate and shorter event-free survival (39, 49). Conversely, NPM1C and FLT3 mutations, while prevalent in AML, have a more complex relationship with prognosis, have certain numbers of NPM1C and FLT3 mutations in C1 and C2.

Of particular interest is the observation that DNMT3A and RUNX1 mutations seem to have a more pronounced influence on ligand-receptor (LR) pair interactions and intercellular communication in the AML microenvironment. This suggests that these mutations might be disrupting the cellular crosstalk essential for hematopoiesis, thereby promoting leukemogenesis. On the other hand, NPM1C and FLT3 mutations, although significant, appear to exert a less direct effect on these LR pairs and cellular communications. This differential impact underscores the importance of considering the individual and combined effects of these mutations. It’s not just their presence, but their influence on cellular networks and communication that might determine disease progression and therapeutic responses. As we move forward, a comprehensive understanding of these mutations, especially in the context of LR pairs, will be vital for tailoring therapeutic strategies and improving prognosis assessments for AML patients.

The low LR.score group displayed a marked potential for sensitivity to immune checkpoint blockade (ICB) treatments. This observation is further supported by the notably T-cell dysfunction score within this group, suggesting an increased probability of a favorable response to anti-PD1/PD-L1 therapies. Immune checkpoints, such as PD-1 and its ligand PD-L1, are crucial modulators of the immune response, and their dysregulation can be leveraged by cancer cells to evade immune surveillance (50–53). The presence and abundance of specific immune cells, especially CD8 T cells and Tregs, could be pivotal in determining the therapeutic outcome of ICB treatments. CD8 T cells are the primary effectors in antitumor immunity, responsible for recognizing and killing cancer cells. The reinvigoration of these cells through ICB has been linked to improved patient outcomes in multiple studies (54, 55). Considering the low LR.score group’s immune cell composition and TIDE score, it is plausible that they would benefit significantly from ICB therapies. Further analysis of the underlying molecular mechanisms revealed that LR pairs such as CXCL12-ITGA4 and HGF-MET, which are significantly downregulated in the low LR.score group, might contribute to the observed T-cell dysfunction. The CXCL12-ITGA4 axis is known to regulate the trafficking and homing of T cells to the bone marrow, and its downregulation could lead to impaired T-cell recruitment and function in the tumor microenvironment (56, 57). Similarly, the HGF-MET pathway has been implicated in promoting T-cell exhaustion through the activation of downstream signaling pathways, such as PI3K/AKT and MAPK/ERK (58, 59), which are involved in maintaining the immunosuppressive tumor microenvironment.

Moreover, the negative correlation between LR.score and T-cell regulatory pathways, including the TGF-beta signaling pathway, suggests that the low LR.score group may have reduced immunosuppressive signaling, further enhancing the potential effectiveness of ICB therapies. TGF-beta is a well-known suppressor of T-cell function, and its downregulation in the low LR.score group could lead to a more favorable immune microenvironment for the activation and expansion of effector T cells in response to ICB.

A higher LR score, indicative of altered ligand-receptor interactions, can potentially impact the finely-tuned signaling pathways that regulate hematopoiesis. These disruptions can further skew the differentiation of hematopoietic stem cells (HSCs) towards the myeloid lineage, leading to an accumulation of blasts and a concomitant decrease in lymphoid cells, especially T cells. The decreased differentiation and maturation of lymphoid progenitors could potentially explain the T-cell dysfunction observed in high LR score patients.

Moreover, the T cell differentiation block, a hallmark of aggressive AML, can be particularly concerning. T cells play a pivotal role in immune surveillance, and their differentiation block can facilitate an immunosuppressive environment, which AML cells exploit for their survival and proliferation. This can lead to a vicious cycle where the increased number of AML blasts further suppresses T cell differentiation, leading to a more aggressive disease phenotype. Considering these intricate biological interactions, it becomes evident that strategies aiming to restore T-cell function and differentiation might be particularly beneficial for AML patients with high LR scores. Immune checkpoint blockade therapies, which have shown promise in rejuvenating exhausted T cells in other malignancies, could potentially rectify the T cell differentiation block and restore the balance in hematopoiesis. This approach, when combined with a deeper understanding of the molecular underpinnings of AML, offers a promising avenue for more targeted and effective therapies for this aggressive malignancy.

Our study also yields with limitations. First and foremost, while our LR.score has shown promise in predicting survival in an independent AML cohort, it is based on retrospective data, which inherently carries potential biases. Prospective validation in diverse patient populations is essential to establish its universal applicability. Moreover, the molecular complexities of AML and its subtypes might not be entirely captured by the LR.score alone. The nuances of individual patient genetics, epigenetics, and the ever-evolving tumor microenvironment might introduce variations not accounted for in our model. Additionally, our study predominantly focuses on T-cell dysfunction and its relationship with the LR.score, potentially overlooking other critical components influencing AML progression. Lastly, while our findings provide a foundation for therapeutic considerations, actual clinical efficacy requires rigorous testing through controlled clinical trials.

The critical ligand-receptor pairs such as ICAM4-ITGA4, CLEC11A-ITGA11, AVP-AVPR1B, along with CXCL12-ITGA4, HGF-MET, IL2-IL2RA, NCAM1-ROBO3, and NLGN1-NRXN2, sheds light on the complexity of leukemic pathophysiology. These genes, through their respective ligand-receptor interactions, are essential in maintaining normal bone marrow function, facilitating precise communication between cellular and extracellular components, and ensuring proper immune function and stress response. The downregulation of ICAM4 and its receptor ITGA4 compromises not just the physical adhesion of hematopoietic cells to the bone marrow stroma but also interrupts intracellular signaling pathways that guide cell fate decisions, which can be a consequence of the leukemic cells outcompeting their normal counterparts for niche occupancy and resources (60–62). Similarly, the diminished expression of CLEC11A and ITGA11, possibly due to epigenetic phenomena like promoter hypermethylation, disrupts not only the proliferation and differentiation of hematopoietic stem cells but also the integrity of the bone marrow’s extracellular matrix—a critical scaffold for cellular interactions and signaling (63, 64). Furthermore, the hormonal regulation of the bone marrow environment, exemplified by the AVP-AVPR1B axis, is crucial in modulating responses to physiological stress and may be disrupted in AML, reflecting a systemic dysregulation of homeostatic mechanisms in response to the leukemic burden (65). Disruptions in cell adhesion molecules like NCAM1 and their interactions with receptors such as ROBO3, along with synaptic adhesion molecules like NLGN1 and their neurexin partners such as NRXN2, suggest a broader disruption of intercellular communication within the bone marrow niche, extending beyond the classical pathways known to be involved in hematopoiesis (66, 67). The downregulation of these critical genes (e.g., CLEC11A, ICAM4, ITGA4, and AVP) and their pathways in AML is not merely indicative of the disease’s pathology but may also serve as negative biomarkers, which could provide valuable insights into the disease’s severity, progression, and responsiveness to treatment. Their expression levels could potentially inform prognostic stratification and therapeutic targeting, underlining the necessity for continued research and development of interventions that can specifically modulate these dysregulated pathways. The intricate interplay of these gene expressions and their associated signaling cascades represents a sophisticated network that, when altered, contributes to the malignant phenotype of AML, emphasizing the importance of a nuanced understanding of these molecular mechanisms in the quest for more effective treatments.

## Conclusion

The high ligand-receptor (LR) scores identified in Acute Myeloid Leukemia (AML) are strongly associated with T-cell dysfunction, underscoring the complex molecular interactions within the disease. Our findings validate the LR.score as a robust prognostic marker that accurately predicts survival outcomes and stratifies patients based on their likely responses to chemotherapy and immunotherapy. Clinically, the LR.score could be instrumental in guiding personalized treatment strategies by identifying patients who may benefit from specific therapies, such as immune checkpoint inhibitors or targeted treatments, based on their LR interaction profiles. This approach not only enhances prognostic precision but also supports the development of tailored therapeutic interventions, paving the way for more effective and individualized management of AML.

## Data Availability

The datasets presented in this study can be found in online repositories. The names of the repository/repositories and accession number(s) can be found in the article/**Supplementary Material.**

## Supplementary material

Supplementary Figure 1
**(A)** Trajectory depiction for each LR pair, with the horizontal axis representing the log value of each lambda and the vertical axis showcasing coefficient values. **(B)** Partial likelihood deviance for each ln(lambda) within the LASSO Cox regression model. **(C)** LASSO Cox coefficients for identified risk and protective LR pairs.

## References

[1] Dohner H Estey EH Amadori S Appelbaum FR Buchner T Burnett AK. Diagnosis and management of acute myeloid leukemia in adults: recommendations from an international expert panel, on behalf of the European LeukemiaNet. Blood. (2010) 115:453–74. doi: 10.1182/blood-2009-07-235358 19880497

[2] Dohner H Weisdorf DJ Bloomfield CD. Acute myeloid leukemia. N Engl J Med. (2015) 373:1136–52. doi: 10.1056/NEJMra1406184 26376137

[3] Stone RM Mandrekar SJ Sanford BL Laumann K Geyer S Bloomfield CD. Midostaurin plus chemotherapy for acute myeloid leukemia with a FLT3 mutation. N Engl J Med. (2017) 377:454–64. doi: 10.1056/NEJMoa1614359 PMC5754190 28644114

[4] Strickland SA Vey N. Diagnosis and treatment of therapy-related acute myeloid leukemia. Crit Rev Oncol Hematol. (2022) 171:103607. doi: 10.1016/j.critrevonc.2022.103607 35101585

[5] Lim JJ Othus M Shaw CM Russell K Halpern AB Appelbaum JS. Time independent factors that predict relapse in adults with acute myeloid leukemia. Blood Cancer J. (2024) 14:5. doi: 10.1038/s41408-023-00954-z 38221523 PMC10788333

[6] Tyner JW Tognon CE Bottomly D Wilmot B Kurtz SE Savage SL. Functional genomic landscape of acute myeloid leukaemia. Nature. (2018) 562:526–31. doi: 10.1038/s41586-018-0623-z PMC6280667 30333627

[7] Cancer Genome Atlas Research N Ley TJ Miller C Ding L Raphael BJ Mungall AJ. Genomic and epigenomic landscapes of adult *de novo* acute myeloid leukemia. N Engl J Med. (2013) 368:2059–74. doi: 10.1056/NEJMoa1301689 PMC3767041 23634996

[8] Miles LA Bowman RL Merlinsky TR Csete IS Ooi AT Durruthy-Durruthy R. Single-cell mutation analysis of clonal evolution in myeloid Malignancies. Nature. (2020) 587:477–82. doi: 10.1038/s41586-020-2864-x PMC7677169 33116311

[9] Papaemmanuil E Gerstung M Bullinger L Gaidzik VI Paschka P Roberts ND. Genomic classification and prognosis in acute myeloid leukemia. N Engl J Med. (2016) 374:2209–21. doi: 10.1056/NEJMoa1516192 PMC4979995 27276561

[10] Bullinger L Dohner K Dohner H. Genomics of acute myeloid leukemia diagnosis and pathways. J Clin Oncol. (2017) 35:934–46. doi: 10.1200/JCO.2016.71.2208 28297624

[11] Narayanan D Pozdnyakova O Hasserjian RP Patel SS Weinberg OK. Effect of DNMT3A variant allele frequency and double mutation on clinicopathologic features of patients with *de novo* AML. Blood Adv. (2021) 5:2539–49. doi: 10.1182/bloodadvances.2021004250 PMC8238486 34100902

[12] Uckelmann HJ Haarer EL Takeda R Wong EM Hatton C Marinaccio C. Mutant NPM1 directly regulates oncogenic transcription in acute myeloid leukemia. Cancer Discovery. (2023) 13:746–65. doi: 10.1158/2159-8290.CD-22-0366 PMC10084473 36455613

[13] Short NJ Nguyen D Ravandi F. Treatment of older adults with FLT3-mutated AML: Emerging paradigms and the role of frontline FLT3 inhibitors. Blood Cancer J. (2023) 13:142. doi: 10.1038/s41408-023-00911-w 37696819 PMC10495326

[14] Shafat MS Oellerich T Mohr S Robinson SD Edwards DR Marlein CR. Leukemic blasts program bone marrow adipocytes to generate a protumoral microenvironment. Blood. (2017) 129:1320–32. doi: 10.1182/blood-2016-08-734798 28049638

[15] Uy GL Aldoss I Foster MC Sayre PH Wieduwilt MJ Advani AS. Flotetuzumab as salvage immunotherapy for refractory acute myeloid leukemia. Blood. (2021) 137:751–62. doi: 10.1182/blood.2020007732 PMC7885824 32929488

[16] Vadakekolathu J Rutella S. Escape from T-cell-targeting immunotherapies in acute myeloid leukemia. Blood. (2024) 143:2689–700. doi: 10.1182/blood.2023019961 PMC11251208 37467496

[17] Kuett A Rieger C Perathoner D Herold T Wagner M Sironi S. IL-8 as mediator in the microenvironment-leukaemia network in acute myeloid leukaemia. Sci Rep. (2015) 5:18411. doi: 10.1038/srep18411 26674118 PMC4682064

[18] Li Z Herold T He C Valk PJ Chen P Jurinovic V. Identification of a 24-gene prognostic signature that improves the European LeukemiaNet risk classification of acute myeloid leukemia: an international collaborative study. J Clin Oncol. (2013) 31:1172–81. doi: 10.1200/JCO.2012.44.3184 PMC3595425 23382473

[19] Herold T Metzeler KH Vosberg S Hartmann L Rollig C Stolzel F. Isolated trisomy 13 defines a homogeneous AML subgroup with high frequency of mutations in spliceosome genes and poor prognosis. Blood. (2014) 124:1304–11. doi: 10.1182/blood-2013-12-540716 24923295

[20] Herold T Jurinovic V Batcha AMN Bamopoulos SA Rothenberg-Thurley M Ksienzyk B. A 29-gene and cytogenetic score for the prediction of resistance to induction treatment in acute myeloid leukemia. Haematologica. (2018) 103:456–65. doi: 10.3324/haematol.2017.178442 PMC5830382 29242298

[21] Metzeler KH Hummel M Bloomfield CD Spiekermann K Braess J Sauerland MC. An 86-probe-set gene-expression signature predicts survival in cytogenetically normal acute myeloid leukemia. Blood. (2008) 112:4193–201. doi: 10.1182/blood-2008-02-134411 PMC2954679 18716133

[22] Wang YH Lin CC Hsu CL Hung SY Yao CY Lee SH. Distinct clinical and biological characteristics of acute myeloid leukemia with higher expression of long noncoding RNA KIAA0125. Ann Hematol. (2021) 100:487–98. doi: 10.1007/s00277-020-04358-y PMC7817567 33225420

[23] Hou R Denisenko E Ong HT Ramilowski JA Forrest ARR. Predicting cell-to-cell communication networks using NATMI. Nat Commun. (2020) 11:5011. doi: 10.1038/s41467-020-18873-z 33024107 PMC7538930

[24] Storey JD. A direct approach to false discovery rates. J R Stat Society: Ser B (Statistical Methodology). (2002) 64:479–98. doi: 10.1111/1467-9868.00346

[25] Wilkerson MD Hayes DN. ConsensusClusterPlus: a class discovery tool with confidence assessments and item tracking. Bioinformatics. (2010) 26:1572–3. doi: 10.1093/bioinformatics/btq170 PMC2881355 20427518

[26] Senbabaoglu Y Michailidis G Li JZ. Critical limitations of consensus clustering in class discovery. Sci Rep. (2014) 4:6207. doi: 10.1038/srep06207 25158761 PMC4145288

[27] Mootha VK Lindgren CM Eriksson KF Subramanian A Sihag S Lehar J. PGC-1alpha-responsive genes involved in oxidative phosphorylation are coordinately downregulated in human diabetes. Nat Genet. (2003) 34:267–73. doi: 10.1038/ng1180 12808457

[28] Subramanian A Tamayo P Mootha VK Mukherjee S Ebert BL Gillette MA. Gene set enrichment analysis: a knowledge-based approach for interpreting genome-wide expression profiles. Proc Natl Acad Sci U S A. (2005) 102:15545–50. doi: 10.1073/pnas.0506580102 PMC1239896 16199517

[29] Liberzon A Birger C Thorvaldsdottir H Ghandi M Mesirov JP Tamayo P. The Molecular Signatures Database (MSigDB) hallmark gene set collection. Cell Syst. (2015) 1:417–25. doi: 10.1016/j.cels.2015.12.004 PMC4707969 26771021

[30] Yu G Wang LG Han Y He QY. clusterProfiler: an R package for comparing biological themes among gene clusters. OMICS. (2012) 16:284–7. doi: 10.1089/omi.2011.0118 PMC3339379 22455463

[31] Newman AM Liu CL Green MR Gentles AJ Feng W Xu Y. Robust enumeration of cell subsets from tissue expression profiles. Nat Methods. (2015) 12:453–7. doi: 10.1038/nmeth.3337 PMC4739640 25822800

[32] Jiang P Gu S Pan D Fu J Sahu A Hu X. Signatures of T cell dysfunction and exclusion predict cancer immunotherapy response. Nat Med. (2018) 24:1550–8. doi: 10.1038/s41591-018-0136-1 PMC6487502 30127393

[33] Hanzelmann S Castelo R Guinney J. GSVA: gene set variation analysis for microarray and RNA-seq data. BMC Bioinf. (2013) 14:7. doi: 10.1186/1471-2105-14-7 PMC3618321 23323831

[34] Mayakonda A Lin DC Assenov Y Plass C Koeffler HP. Maftools: efficient and comprehensive analysis of somatic variants in cancer. Genome Res. (2018) 28:1747–56. doi: 10.1101/gr.239244.118 PMC6211645 30341162

[35] Geeleher P Cox N Huang RS. pRRophetic: an R package for prediction of clinical chemotherapeutic response from tumor gene expression levels. PloS One. (2014) 9:e107468. doi: 10.1371/journal.pone.0107468 25229481 PMC4167990

[36] Mariathasan S Turley SJ Nickles D Castiglioni A Yuen K Wang Y. TGFbeta attenuates tumour response to PD-L1 blockade by contributing to exclusion of T cells. Nature. (2018) 554:544–8. doi: 10.1038/nature25501 PMC6028240 29443960

[37] Liu Y He M Wang D Diao L Liu J Tang L. HisgAtlas 1.0: a human immunosuppression gene database. Database (Oxford). (2017) 2017:bax094. doi: 10.1093/database/bax094 31725860 PMC7243927

[38] Ley TJ Ding L Walter MJ McLellan MD Lamprecht T Larson DE. DNMT3A mutations in acute myeloid leukemia. N Engl J Med. (2010) 363:2424–33. doi: 10.1056/NEJMoa1005143 PMC3201818 21067377

[39] Mendler JH Maharry K Radmacher MD Mrozek K Becker H Metzeler KH. RUNX1 mutations are associated with poor outcome in younger and older patients with cytogenetically normal acute myeloid leukemia and with distinct gene and MicroRNA expression signatures. J Clin Oncol. (2012) 30:3109–18. doi: 10.1200/JCO.2011.40.6652 PMC3732007 22753902

[40] Bohme M Kayser S. Immune-based therapeutic strategies for acute myeloid leukemia. Cancers (Basel). (2021) 14:105. doi: 10.3390/cancers14010105 35008269 PMC8744886

[41] Witkowski MT Lasry A Carroll WL Aifantis I. Immune-based therapies in acute leukemia. Trends Cancer. (2019) 5:604–18. doi: 10.1016/j.trecan.2019.07.009 PMC6859901 31706508

[42] Sun Y Wang R Xie S Wang Y Liu H. A novel identified necroptosis-related risk signature for prognosis prediction and immune infiltration indication in acute myeloid leukemia patients. Genes (Basel). (2022) 13:1837. doi: 10.3390/genes13101837 36292722 PMC9602382

[43] Chen Y You S Li J Zhang Y Kokaraki G Epstein E. Follicular helper T-cell-based classification of endometrial cancer promotes precise checkpoint immunotherapy and provides prognostic stratification. Front Immunol. (2021) 12:788959. doi: 10.3389/fimmu.2021.788959 35069566 PMC8777298

[44] Huang X Chen C Xu Y Shen L Chen Y Su H. Infiltrating T-cell abundance combined with EMT-related gene expression as a prognostic factor of colon cancer. Bioengineered. (2021) 12:2688–701. doi: 10.1080/21655979.2021.1939618 PMC8806648 34180352

[45] Chen Y Chen D Wang Q Xu Y Huang X Haglund F. Immunological classification of pancreatic carcinomas to identify immune index and provide a strategy for patient stratification. Front Immunol. (2021) 12:719105. doi: 10.3389/fimmu.2021.719105 35111149 PMC8801451

[46] Cheng WY Li JF Zhu YM Lin XJ Wen LJ Zhang F. Transcriptome-based molecular subtypes and differentiation hierarchies improve the classification framework of acute myeloid leukemia. Proc Natl Acad Sci U S A. (2022) 119:e2211429119. doi: 10.1073/pnas.2211429119 36442087 PMC9894241

[47] Ribeiro AF Pratcorona M Erpelinck-Verschueren C Rockova V Sanders M Abbas S. Mutant DNMT3A: a marker of poor prognosis in acute myeloid leukemia. Blood. (2012) 119:5824–31. doi: 10.1182/blood-2011-07-367961 22490330

[48] Gaidzik VI Schlenk RF Paschka P Stolzle A Spath D Kuendgen A. Clinical impact of DNMT3A mutations in younger adult patients with acute myeloid leukemia: results of the AML Study Group (AMLSG). Blood. (2013) 121:4769–77. doi: 10.1182/blood-2012-10-461624 23632886

[49] Tang JL Hou HA Chen CY Liu CY Chou WC Tseng MH. AML1/RUNX1 mutations in 470 adult patients with *de novo* acute myeloid leukemia: prognostic implication and interaction with other gene alterations. Blood. (2009) 114:5352–61. doi: 10.1182/blood-2009-05-223784 19808697

[50] Topalian SL Drake CG Pardoll DM. Immune checkpoint blockade: a common denominator approach to cancer therapy. Cancer Cell. (2015) 27:450–61. doi: 10.1016/j.ccell.2015.03.001 PMC4400238 25858804

[51] Pardoll DM. The blockade of immune checkpoints in cancer immunotherapy. Nat Rev Cancer. (2012) 12:252–64. doi: 10.1038/nrc3239 PMC4856023 22437870

[52] Zhang Y Chen Y Papakonstantinou A Tsagkozis P Linder-Stragliotto C Haglund F. Evaluation of PD-L1 expression in undifferentiated pleomorphic sarcomas, liposarcomas and chondrosarcomas. Biomolecules. (2022) 12:292. doi: 10.3390/biom12020292 35204793 PMC8961782

[53] Xu Y Chen D Shen L Huang X Chen Y Su H. Identification and mechanism of the PD-1/PD-L1 genomic signature SORL1 as protective factor in bladder cancer. Front Genet. (2021) 12:736158. doi: 10.3389/fgene.2021.736158 34976002 PMC8716752

[54] Schreiber RD Old LJ Smyth MJ. Cancer immunoediting: integrating immunity’s roles in cancer suppression and promotion. Science. (2011) 331:1565–70. doi: 10.1126/science.1203486 21436444

[55] Tumeh PC Harview CL Yearley JH Shintaku IP Taylor EJ Robert L. PD-1 blockade induces responses by inhibiting adaptive immune resistance. Nature. (2014) 515:568–71. doi: 10.1038/nature13954 PMC4246418 25428505

[56] Ennis S Conforte A O’Reilly E Takanlu JS Cichocka T Dhami SP. Cell-cell interactome of the hematopoietic niche and its changes in acute myeloid leukemia. iScience. (2023) 26:106943. doi: 10.1016/j.isci.2023.106943 37332612 PMC10275994

[57] Shi Y Riese DJ 2nd Shen J. The role of the CXCL12/CXCR4/CXCR7 chemokine axis in cancer. Front Pharmacol. (2020) 11:574667. doi: 10.3389/fphar.2020.574667 33363463 PMC7753359

[58] Liu ZL Chen HH Zheng LL Sun LP Shi L. Angiogenic signaling pathways and anti-angiogenic therapy for cancer. Signal Transduct Target Ther. (2023) 8:198. doi: 10.1038/s41392-023-01460-1 37169756 PMC10175505

[59] Kim J Lee TS Lee MH Cho IR Ryu JK Kim YT. Pancreatic cancer treatment targeting the HGF/c-MET pathway: the MEK inhibitor trametinib. Cancers (Basel). (2024) 16:1056. doi: 10.3390/cancers16051056 38473413 PMC10930669

[60] Li M Ye J Xia Y Li M Li G Hu X. METTL3 mediates chemoresistance by enhancing AML homing and engraftment via ITGA4. Leukemia. (2022) 36:2586–95. doi: 10.1038/s41375-022-01696-w PMC9613467 36266324

[61] Kohnke T Liu X Haubner S Bucklein V Hanel G Krupka C. Integrated multiomic approach for identification of novel immunotherapeutic targets in AML. biomark Res. (2022) 10:43. doi: 10.1186/s40364-022-00390-4 35681175 PMC9185890

[62] Lee G Spring FA Parsons SF Mankelow TJ Peters LL Koury MJ. Novel secreted isoform of adhesion molecule ICAM-4: potential regulator of membrane-associated ICAM-4 interactions. Blood. (2003) 101:1790–7. doi: 10.1182/blood-2002-08-2529 12406883

[63] Swanson AJ Rogowski VJ Bishop JA Walker DM Roxas GM Raimondi SL. CLEC11A methylation is correlated to AML subtypes and cytogenetic risk factors but not patient demographics. PloS One. (2024) 19:e0300477. doi: 10.1371/journal.pone.0300477 38466706 PMC10927138

[64] Yin C Zhang J Guan W Dou L Liu Y Shen M. High expression of CLEC11A predicts favorable prognosis in acute myeloid leukemia. Front Oncol. (2021) 11:608932. doi: 10.3389/fonc.2021.608932 33747924 PMC7966831

[65] Freiwan A Zoine JT Crawford JC Vaidya A Schattgen SA Myers JA. Engineering naturally occurring CD7- T cells for the immunotherapy of hematological Malignancies. Blood. (2022) 140:2684–96. doi: 10.1182/blood.2021015020 PMC9935551 35914226

[66] Cai Z Wei J Chen Z Wang H. High ROBO3 expression predicts poor survival in non-M3 acute myeloid leukemia. Exp Biol Med (Maywood). (2021) 246:1184–97. doi: 10.1177/1535370220988246 PMC8142108 33541130

[67] Sasca D Szybinski J Schuler A Shah V Heidelberger J Haehnel PS. NCAM1 (CD56) promotes leukemogenesis and confers drug resistance in AML. Blood. (2019) 133:2305–19. doi: 10.1182/blood-2018-12-889725 30814062

