# Supplementary figures and images for: Characterization of ligand-receptor pair in acute myeloid leukemia: a scoring model for prognosis, therapeutic response, and T cell dysfunction
Source: Front Oncol. 2024 Oct 17;14:1473048. doi: 10.3389/fonc.2024.1473048 (PMC11525004; doi:10.3389/fonc.2024.1473048)

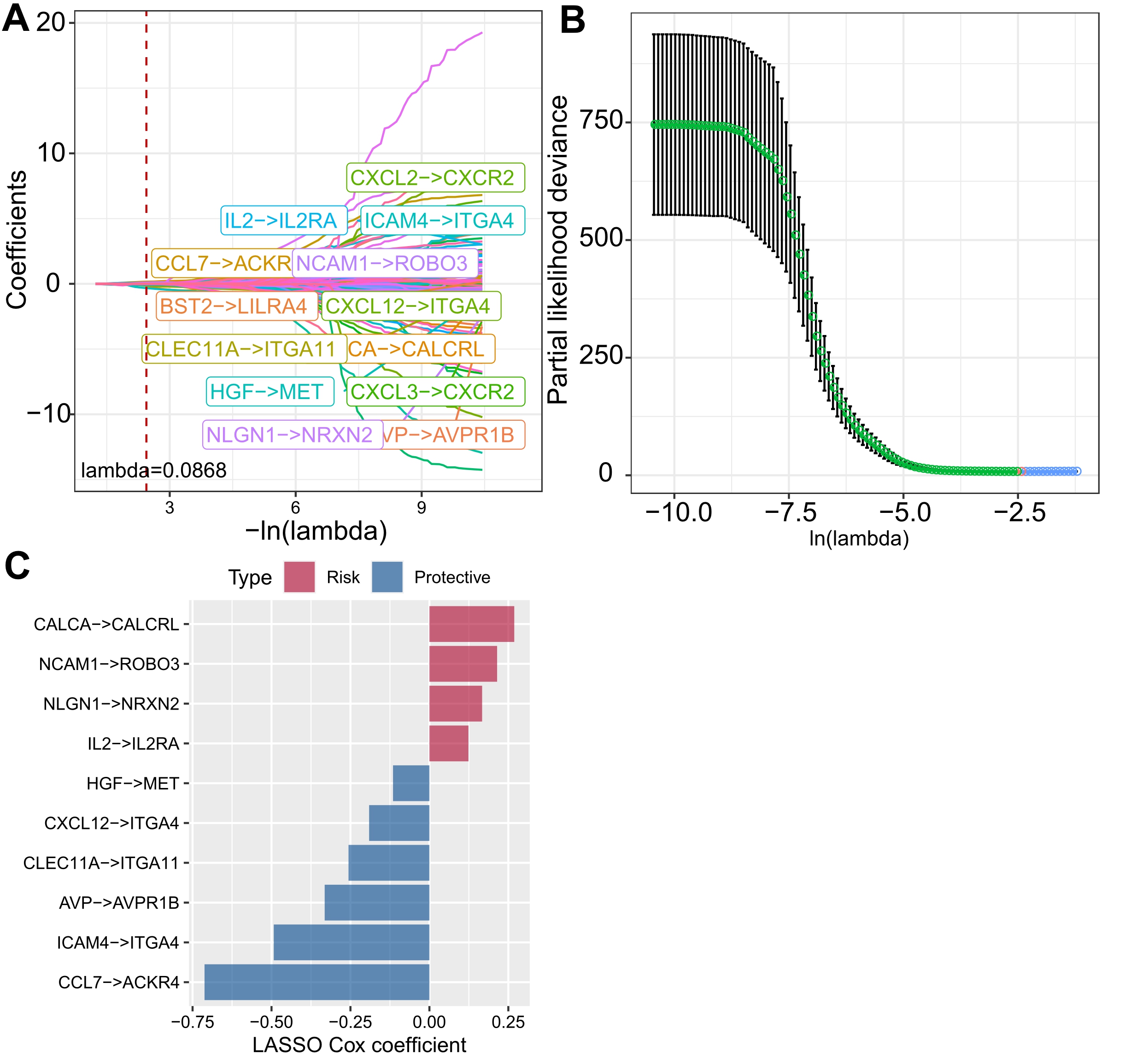

Supplement: Supplementary Figure 1 — (A) Trajectory depiction for each LR pair, with the horizontal axis representing the log value of each lambda and the vertical axis showcasing coefficient values. (B) Partial likelihood deviance for each ln(lambda) within the LASSO Cox regression model. (C) LASSO Cox coefficients for identified risk and protective LR pairs. [file Image1.jpeg]
